# Patient classification using single-cell RNA-sequencing data with deep learning convolutional neural networks

**DOI:** 10.3389/fbinf.2026.1919127

**Published:** 2026-09-29

**Authors:** Anastasis Oulas, Nestoras Karathanasis, Theodosios Lampadarios, Raphael Antartis, George M. Spyrou

**Affiliations:** Bioinformatics Department, The Cyprus Institute of Neurology and Genetics, Nicosia, Cyprus

**Keywords:** convolutional neural networks, deep learning, gene expression, patient classification, single-cell RNA sequencing (scRNA-seq)

## Abstract

**Introduction:**

Single-cell RNA sequencing (scRNA-seq) enables gene expression profiling at the resolution of individual cells, revealing cellular heterogeneity that is obscured in bulk RNA-seq. While this offers a powerful lens to study disease-specific changes, leveraging scRNA-seq data for patient phenotype classification remains challenging due to limited sample numbers, cell count variability, high dimensionality, and dependence on cell-type annotations. Existing tools attempt to address these issues using probabilistic models or attention mechanisms, but face limitations in scalability, interpretability, or representational power.

**Methods:**

In this work, we introduce DeepCelllify, a novel deep learning-based framework that harnesses convolutional neural networks (CNNs) to classify patients using their individual cell expression profiles. Our approach converts tabular gene expression data into image-like representations, enabling CNNs to learn spatial feature relationships between genes. Each cell is treated as an independent input labeled by its originating patient’s condition (e.g., disease or control), allowing us to sidestep the limitations posed by small patient cohorts and heterogeneous cell counts. The DeepCelllify pipeline includes a transformation module for data preprocessing and a classification module, culminating in CNN models trained through cross-validation to differentiate between disease states based on single-cell inputs.

**Results:**

Our method demonstrates competitive performance compared to state-of-the-art approaches, with added benefits in scalability, interpretability, and practical applicability. DeepCelllify further addresses critical bottlenecks in scRNA-seq-based phenotype prediction, including overfitting from low patient counts, challenges in feature matrix construction, and dimensionality issues. Additionally, our method provides interpretability by assigning weights to individual cells, helping to down-weight misclassified or noisy cells.

**Discussion:**

This work introduces deep learning framework for patient phenotype prediction from scRNA-seq data, with potential applications in precision medicine and biomarker discovery.

## 1 Introduction

Single-cell RNA sequencing (scRNA-seq) provides an additive resolution down to the cellular level that was previously not available from traditional “Bulk” RNA sequencing experiments. ScRNA-seq analysis generates clusters of cells that are grouped together due to their similar expression profiles. These clusters define specific cell types and provide scRNA-seq data with increased resolution in comparison to bulk-RNA-Seq. This has apparent advantages when studying disease phenotypes because researchers can now narrow down the specific cell types that are affected by a given disease and perform more targeted, specialized analyses. By examining genes within a specific cell type, we can infer a unique expression pattern that defines that cell. However, it is often unclear which cell types may be more informative for a specific disease under study, both from a molecular, as well as a diagnostic perspective.

Currently very little work has been done on the use of scRNA-seq data for the automatic classification of patients’ phenotypes. This can be attributed to the following reasons. (i) the fact that scRNA-seq datasets are normally characterized by a small number of patient samples in accord to the high cost of the technology. Concurrently, this prevents the adequate training of classifiers leading to undertrained model construction or overfitting. (ii) Another issue with the usability of scRNA-seq data for classification methods is the variable number of cells across collected samples from the same dataset, rendering these types of datasets inapplicable for model training. (iii) In addition, the large number of cells (often hundreds of thousands) and high-dimensional gene expression data in an individual sample contribute towards the “curse of dimensionality” which is a common phenomenon when analysing this type of data. This makes the creation of machine learning models challenging and the prediction results are often hard to justify. (iv) Dependence of accurate cell type identification which might be biased or unavailable. Most scRNA-seq analysis starts with detecting cell types using unsupervised clustering, followed by cell-type annotations based on marker genes. Patients’ phenotypes are then predicted by distributions of cell types or identifying specific cell types. However, accurate cell type identifications are affected by the proper clustering resolution for a given sample, and the marker gene information that might be suboptimal or missing. Therefore, many existing scRNA-seq analysis methods even require users to provide the number of cell types, which is unknown before analysis, or set up a universal value to provide the corresponding analysis results.

Available tools for patient phenotype prediction using scRNA-seq data include CloudPred (He et al., 2022) that models the individual points as samples from a mixture of Gaussians, probabilistically assigns points to clusters, then estimates prevalence of the subpopulations and uses it to predict the phenotype of the patient. scPheno (Zeng et al., 2022) constructs gene expression profiles by a joint distribution of cell states and disease phenotypes based on a deep generative probabilistic model, and feeds the distribution as the predictive features to support vector machine for the phenotype prediction. Both methods assume cell populations follow simple bell-shaped (Gaussian) distributions. But in reality, cell populations can be highly complex, skewed, multi-modal, or nonlinear. So, this assumption may in fact oversimplify biology, reduce prediction accuracy, miss rare or transitional cell states. Even though these methods can train on small datasets, their relatively simple architectures (e.g., Gaussian mixtures, SVMs) and lack of deep learning algorithms, limit their capacity to capture the complex, high-dimensional structure of scRNA-seq data (He et al., 2022; Zeng et al., 2022).

Another recently developed tool ProtoCell4P (Xiong et al., 2023) proposed an interpretable Prototype-based and Cell-informed model for patient classification tasks that leverages the knowledge of scRNA-seq data to predict individual phenotypes, where the “prototypes” are representatives of cells. These prototypes are defined by a cell embedding module which encodes the cells into the latent space and learns to distinguish between a group of cell prototypes that can be representatives of cell subpopulations. Afterwards, a classification module adaptively evaluates the relevance of prototypes in the classification for each cell and combines the prototype-related information from all cells to make the final prediction. While ProtoCell4P provides interpretable prototype-based representations, however its reliance on predefined cell prototypes and latent embeddings may limit its ability to capture complex nonlinear gene interactions and rare cellular states.

Recently the ScRAT tool (Mao et al., 2024), provides a phenotype prediction framework that can learn from limited numbers of scRNA-seq samples with minimal dependence on cell-type annotations. Compared to other available scRNA-seq analysis algorithms (described above) that model gene expression profiles of different cell clusters by separate Gaussian distributions (He et al., 2022; Zeng et al., 2022; He et al., 2022; Zeng et al., 2022), ScRAT, utilizes the attention mechanism to measure interactions between cells as their correlations, or attention weights. For each cell, ScRAT incorporates all of its interaction patterns and attention weights to establish its connections with the corresponding phenotypes. Secondly, it introduces a mix-up module in its framework as a data augmentation approach to mitigate the potential overfitting issue caused by the high model complexity together with the very limited number of labelled samples. Lastly, ScRAT establishes the connection between the input (cells) and the output (phenotypes) of the Transformer model simply using the attention weights. ScRAT hence selects cells containing the most discriminative information to specific phenotypes, or critical cells, using their attention weights. While ScRAT leverages attention mechanisms to model cell–cell interactions and identify phenotype-relevant cells, its reliance on Transformer architectures introduces challenges related to computational scalability, overfitting, and interpretability. Attention weights may not correspond to true biological interactions, and the use of mix-up augmentation can introduce biologically unrealistic signals.

In this work we capitalize on the large number of cells available for scRNA-seq experiments (traditionally from 1000 to 5,000 cells per sample) in order to employ deep learning Convolutional Neural Networks (CNNs) to classify patients by way of their individual cell expression profiles.

Our methodology (DeepCellify) is characterized by two components. A *transformation* component and a *classification* component (see Figure 1). We treat each individual cell as a separate entity allowing the cell to inherit the class label from the individual patient sample by which it was derived from (e.g., disease stage or control). The notion of classifying patients into respective disease or control conditions by way of classifying their individual cells (as separate classification entities) without prior feature engineering or extraction, as a means of performing computational diagnosis has not previously been explored. This represents a distinctive feature of DeepCellify, enabling the classification of individual cells into different signal-associated disease stages and/or control conditions. As many cells may not actually contain disease-related signals, this characteristic permits for the segregation of cells into inferred/putative disease-related categories and hence, allowing for the more accurate molecular characterization of their expression profiles. Another novelty in the DeepCellify methodology, includes the transformation of tabular data into images for deep learning with CNNs using the IGTD tool (Zhu et al., 2021). CNNs are inspired by visual neuroscience and possess key features that exploit the properties of natural signals, including local connections in receptive field, parameter sharing via convolution kernel, and hierarchical feature abstraction through pooling and multiple layers (LeCun et al., 2015). These features make CNNs suitable for analysing data with spatial or temporal dependencies between components (Arel et al., 2010; Ismail Fawaz et al., 2019). In order to harness the power of CNNs for scRNA-seq tabular data, the order of features can be rearranged in a 2-D space to explicitly represent relationships between features (i.e., genes), such as feature categories or similarities (Sharma et al., 2019; Bazgir et al., 2020). This motivates the transformation of tabular data into images, from which CNNs can learn and utilize the feature relationships to improve the prediction performance as compared with models trained on tabular data. We then perform a cross-validation training procedure using CNNs, whereby our CNN models learn to distinguish between cells originating from different disease or control patient samples.

**FIGURE 1:**
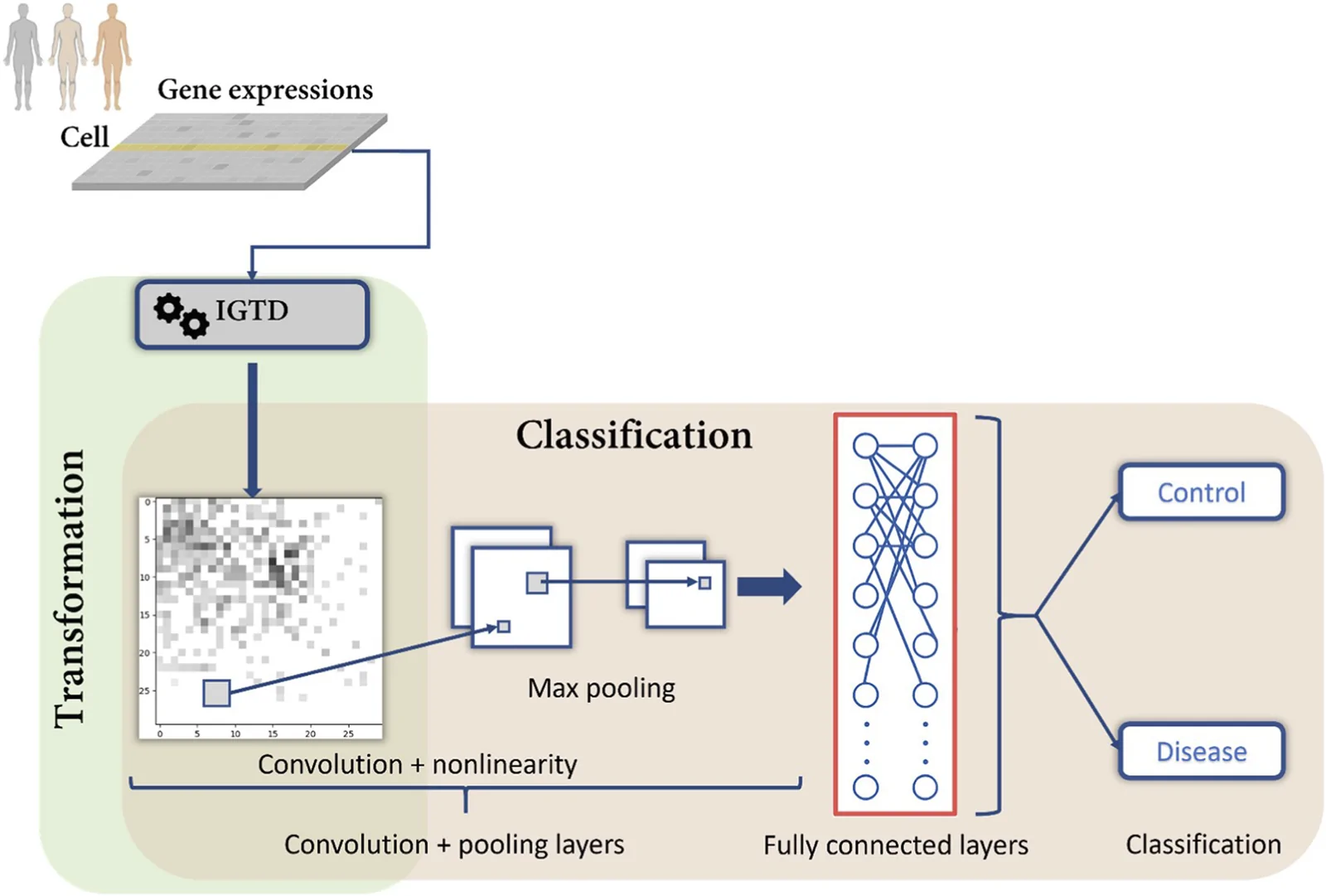
Flow chart showcasing the DeepCellify methodology.

Our approach overcomes most of the limitations mentioned above for other tools. Compared to ProtoCell4P it achieves: 1) learning hierarchical feature representations directly from the data, 2) global structure capturing via convolution + pooling, 3) efficient scaling with large datasets using batch processing and GPUs. In addition, compared to ScRAT, it attains: 1) stronger inductive biases, 2) improved scalability, and the ability to capture structured local patterns, 3) potentially more robust and interpretable representations. DeepCellify also bypasses other limitations that hinder the use of scRNA-seq data in patient’s phenotype diagnostics by: (i) Overcoming issues of statistical significance caused by the small number of patient samples (often less than 100); by utilizing individual cells (often in the hundreds of thousands) as a classification entity. (ii) Mitigating issues caused by the variable number of cells in scRNA-seq datasets such as, difficulties in generating an expression matrix of samples (genes vs. cells across samples) for classification. Our approach performs classification without the need for such a matrix. (iii) Overcoming the curse of dimensionality, a common issue faced when analysing bulk RNA-Seq data, by replacing the conventionally low number of patient samples with thousands of cells. (iv) Our approach still remains depended on the basic scRNA-seq analysis steps such as clustering. However, the fitting/training of our CNN models using individual cell expression data, allows for the model to assign weights to individual cells. Thus, through this way assigning lower weights to those cells that do not contribute towards improving the prediction accuracy for our models.

Finally, we compare patient classification performance of DeepCellify with the latest line of tools (i.e. ScRAT tool (Mao et al., 2024), CloudPred (He et al., 2022), and others) in order to gain a comparison with the state of the art. Something not previously shown by other studies (to the best of our knowledge) is the performance of patient classifiers based on scRNA-seq data in a diagnostic scenario. To achieve this, our trained models were used to classify all cells obtained from a scRNA-seq experiment from an individual patient (test patient sample) using a leave-one-out cross validation (LOOCV) procedure. A diagnosis is obtained by taking in consideration the class label that is given by our models to the majority of the cells in the test patient sample(s).

## 2 Materials and methods

### 2.1 Datasets

Our experiments include 2 datasets (see Table 1).

**TABLE 1:** Datasets used as case studies to train and evaluate DeepCellify.

Datasets	Source	Task	Size	Classes
COVID	(Stephenson et al., 2021)	Disease diagnosis	COVID (n = 102) Non-Covid (n = 41)	COVID vs. Healthy
Multiple Myeloma (MM)	(Dang et al., 2023)	(i) Identification of aymptomatic early stages of the disease (ii) Prediction of disease progression	MGUS (n = 21) SMM (n = 21) NDMM (n = 7) NBM (n = 4)	(i) asymptomatic (MGUS and SMM) vs. symptomatic (NDMM) (ii) MGUS vs. SMM

For the MM dataset we perform 2 classification tasks: (i) identifying non-symptomatic early stages of the disease (MGUS and SMM) vs. symptomatic (NDMM) as well as (ii) predicting disease progression from MGUS to SMM. For the COVID datasets, a single classification task was performed to distinguish between COVID vs. healthy patient samples.

### 2.2 Single-cell RNA-Seq basic analysis

The basic analysis of the RNA-Seq datasets begins by performing scRNA-seq analysis using SEURAT (ver. 5.0.1) (Hao et al., 2021). We utilized standardized analysis pipelines following best practices as described in the SEURAT web page (https://satijalab.org/seurat/index.html). The basic analysis includes, pre-processing, normalization, sample integration using anchors, dimensionality reduction via Principal Component Analysis (PCA), nearest-neighbor graph construction, clustering and detection of 2000 most variable genes (default value according to the best practices recommended by the SEURAT analysis pipeline). Cluster-specific marker genes were identified using the FindAllMarkers function in the SEURAT package, which performs differential expression analysis to determine genes significantly enriched in each cell cluster. The most significantly upregulated marker genes from each cluster were selected and used as input for cell-type annotation. Functional annotation was performed using the enrichR package by querying the CellMarker and PanglaoDB reference databases. Cluster identities were assigned based on the highest-scoring (according to P-value) and biologically relevant cell-type annotations returned by these databases. Custom-made scripts were used to perform code-embedded connections to enrichR via the corresponding R package (enrichR ver. 3.1) (Xie et al., 2021).

### 2.3 The DeepCellify methodology pipeline

#### 2.3.1 Patient information

For each patient, there is a class label that indicates the phenotype of the patient (e.g., NDMM, COVID-19, healthy control). Each patient comprises a collection of cells representing distinct cell types, along with the expression profiles of selected most highly variable genes across these cells (see Figure 1).

#### 2.3.2 Tabular to image conversion (transformation component)

We utilize the Image Generator for Tabular Data (IGTD) algorithm (Zhu et al., 2021) which assigns each feature to a pixel in the image. According to the assignment, an image is generated for each data sample, in our case individual cells, in which the pixel intensity reflects the value of the corresponding feature (gene) in the cell (see Figure 1; Supplementary Figure S1). The algorithm searches for an optimized assignment of genes to pixels by minimizing the difference between the ranking of pairwise distances between genes and the ranking of pairwise distances between the assigned pixels. The distances between pixels are calculated based on their coordinates in the image. The pairwise distances between genes and consequently pixels, are calculated according to a distance measure, specifically Pearson’s Correlation and Manhattan distance respectively. Minimizing the difference between the two rankings assigns genes with similar expression to neighbouring pixels and genes with dissimilar expression to pixels that are far apart. This tabular to image conversion allows each cell to be represented by a unique image where the preselected most highly variable genes are concatenated into 30 × 30-pixel images. Since the image encodes relational structure of genes in our samples (cells), rather than raw values which may be subject to batch effects, the images (cells) become directly comparable.

#### 2.3.3 Cell and patient sample classification (classification component)

Classification is performed by utilizing the images (cells), generated from the transformation component, to train CNNs (see Figure 1, Supplementary Material section on hyperparameter tuning). The optimized CNN architecture utilized by DeepCellify is displayed as a flowchart (see Figure 2). The classification component is written exclusively in R using the tensorflow and keras libraries. Full code is available at https://github.com/aoulas/DeepCellify.

**FIGURE 2:**
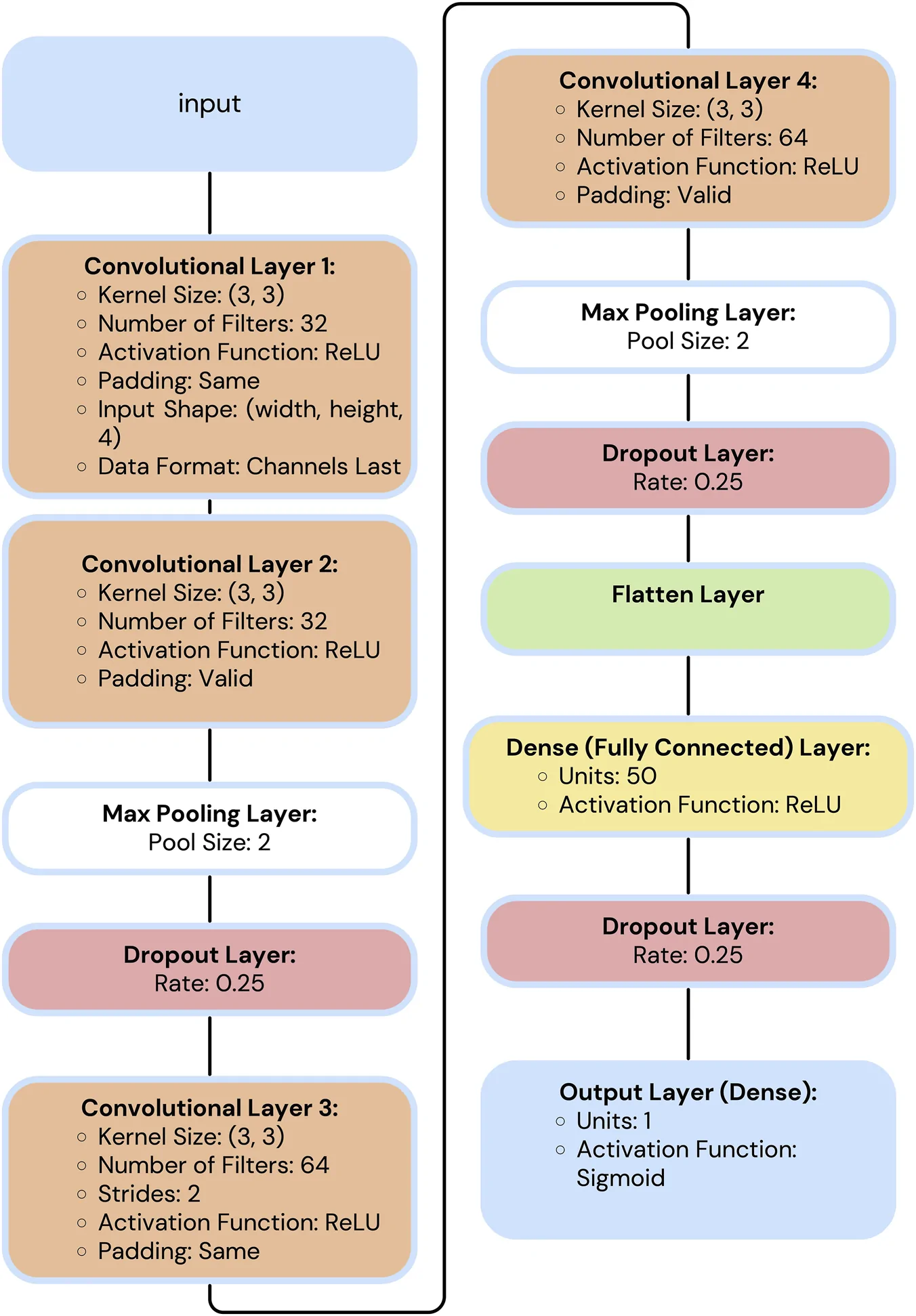
Flowchart of DeepCellify CNN optimized architecture. The model was compiled using the Adam optimizer and binary cross-entropy loss function. Performance was evaluated using multiple metrics, including AUC, recall, specificity, and precision.

#### 2.3.4 Training and cross validation testing

Training is performed by splitting the training data (at the cellular level) using different ratios (50% and 80%), whereby 50% or 80% of the data is retained for training and 50% or 20% for validation respectively. Model(s) optimization is performed by minimization of the loss function (L) between train and validation sets. The model was compiled using the Adam optimizer. Performance was evaluated using multiple metrics, including area under the ROC curve (AUC), recall, specificity, and precision. These metrics were monitored during training to assess classification performance across both positive and negative classes. In addition, an early stopping function is employed to ensure that overfitting does not occur. During training, we evaluate the ratio between validation and training loss (*r = L*_*val*_ */L*_*train*_); when this ratio exceeds 1, the model is considered to exhibit signs of overfitting and training is terminated. Early stoppage is also performed when the validation AUC (val_auc) does not exhibit a significant change (min_delta = 0.01) for 10 consecutive epochs. Testing of our models is performed using a leave-one-out cross validation procedure (LOOCV). A LOOCV procedure was employed whereby CNN models were generated by removing the cells belonging to one patient sample (left-out sample) and training on the cells from the remaining patients. The trained models are then used to classify each individual cell attributed to the left-out patient sample. This process is repeated iteratively until all patients are classified to the category which is defined by the majority of their cellular predictions (majority voting) (see Figure 3). For comparison reasons other aggregation strategies such as probability voting and confidence-based voting were also taken in consideration. We revert to solving multiple binary classification problems (i.e., MGUS|SMM vs. NDMM, MGUS vs. SMM and COVID-19 vs. Healthy) depending on the dataset, as we observed that this approach obtained optimal prediction accuracy results compared to multi-class classification tasks (data not shown). For each dataset, we report both: 1) the average area under the curve (AUC) across the training/validation procedure as defined by Equation 1 (also see Supplementary Table S1; Supplementary Figure S2). This analysis reports results at the cellular level). 2) The overall testing LOOCV performance in the form of sensitivity, specificity, Mathew’s Correlation as well as AUC (the analysis reports results at the patient level). Some extra training steps were performed in order to compare with other published methodologies and tools. Specifically, we followed the same train/validation procedure reported *Mao et al* (Mao et al., 2024). Namely, we performed 100 random train/validation splits across 50% and 80% training ratios (as performed by the authors in the publication), and report our train/validation performance of our best models. As by default DeepCellify calculates training performance at the cellular level, a shift to patient sample level was performed using mean probabilities obtained for cellular predictions.

$${\text{AvePerTrain}}_{\text{Val}}=\text{Sum}\left[\text{valAUCi}\right]/N$$ (1)

Where N is the number of patients in the datasets.

**FIGURE 3:**
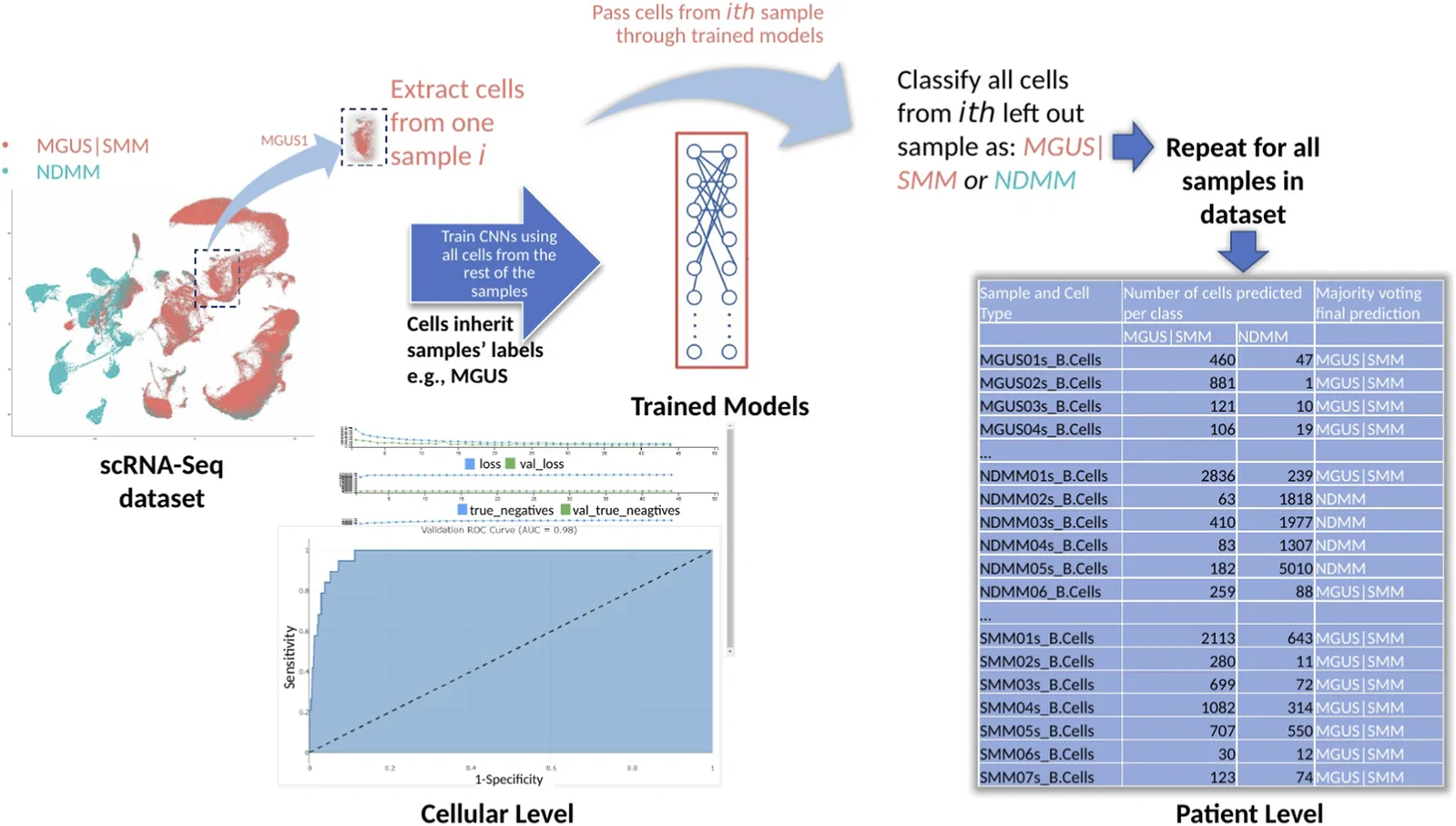
LOOCV overview and majority voting classification based on cell predictions.

### 2.4 Omitting tabular to image conversion step - Ablation study

In order to validate one of the key aspects of our methodology, namely the transformation of tabular to image conversion using IGTD, we proceeded to perform an ablation study using an identical neural network architecture without including the IGTD component. We implemented a feed-forward artificial neural network (ANN) in R using the Keras and TensorFlow libraries. We constructed the network with an input layer, a fully connected hidden layer comprising 64 neurons and employing a rectified linear unit (ReLU) activation function, a dropout layer (dropout rate = 0.2) for regularization, and a softmax output layer for multi-class classification. We trained the model using the RMSprop optimization algorithm with a learning rate of 0.0001 and optimized the categorical cross-entropy loss function. During training, we monitored predictive performance using classification accuracy and the area under the receiver operating characteristic curve (AUC). We trained the network for up to 100 epochs using a batch size of 256 and applied early stopping with a patience of 10 epochs based on validation-set performance to reduce overfitting. We evaluated model performance using a leave-one-out (patient) cross-validation framework. In each iteration, we designated one patient as the independent test case and used the remaining patients for model development. We then randomly partitioned the development patients into training (50%) and validation (50%) subsets using stratified sampling to preserve class proportions. To avoid information leakage, all single-cell observations from a given patient were assigned to the same subset, ensuring that no patient contributed cells to both the training and validation datasets. We selected the final model from the epoch that achieved the best validation performance before early stopping terminated training.

### 2.5 Classification of patient sample cells and extraction of biologically meaningful disease-related molecular signatures

DeepCellify assigns the patient-level phenotype label to individual cells, assuming that every cell inherits the patient’s label. While this strategy greatly increases the training sample size, it may also introduce weak supervision because many cells may not actually contain disease-related signals. In order to show that our method’s cellular-level classification is meaningful and does not hinder classification, we utilized the MM dataset specifically for the MGUS and SMM vs. NDMM binary classification problem. After classifying all patient B-Cells into MGUS and SMM vs. NDMM we proceeded to extract the expression values for the 2000 genes for each respective patient category. We then perform analysis of differentially expressed genes (DEG) using the Wilcox two-sided test. All significant genes were further used for pathway enrichment analysis using 4 different databases: Gene Ontology (GO), Kyoto Encyclopedia of Genes and Genomes (KEGG), REACTOME and Molecular Signatures Database (MSigDB) Hallmark Collection.

### 2.6 Mapping Grad-CAM activations to biologically informative genes – “from pixels to genes”

To interpret model predictions in a biologically meaningful manner, we established a pipeline to map Gradient-weighted Class Activation Mapping (Grad-CAM) (Selvaraju et al., 2017) activation heatmaps back to gene-level features derived from IGTD. Grad-CAM is a technique that highlights the regions of an input (e.g., an image) that most influence a neural network’s prediction by using gradients flowing into the final convolutional layer. As mentioned above, IGTD was used to transform high-dimensional tabular gene expression data into structured 2D image representations, where each pixel corresponds to a feature placement optimized to preserve pairwise feature relationships. The IGTD output consisted of two serialized files (.pkl): a primary file containing the normalized tabular data, generated image tensors, and sample identifiers, and an auxiliary file containing feature ranking matrices and coordinate mappings. These files were loaded using Python (v3.9.5) with the “pickle”, “numpy”, and “pandas” libraries. Custom Python functions were developed to reconstruct the mapping between image pixels and original gene features. This was achieved by extracting the final feature ordering from the IGTD ranking matrix and aligning it with pixel coordinates provided in the auxiliary file. The resulting mapping table linked each pixel position to a specific gene identifier. Grad-CAM heatmaps, generated per sample (cell) and exported as tab-delimited text files, were then integrated with this mapping. For each heatmap, pixel intensities were matched to corresponding genes using the reconstructed mapping. Gene-level importance scores were computed by aggregating activation values across pixels assigned to each gene. To enable robust analysis across samples, the mapping function was applied iteratively to all heatmaps within a directory, and results were concatenated into a unified dataset. Downstream statistical analyses, including frequency of gene occurrence and aggregation of importance measures were performed. This approach enables the translation of spatial activation patterns into interpretable gene-level insights, facilitating biological interpretation of deep learning models trained on omics data.

For comparison reasons we also utilized SHapley Additive exPlanations (SHAP) as and additional interpretation strategy. Similar heatmaps were generated per sample (cell) using SHAP and an identical mapping approach was followed as described above for the Grad-CAM heatmaps.

## 3 Results

### 3.1 Training and leave-one-out cross validation performance

#### 3.1.1 MM dataset

In order to simulate the prediction of a single patient in a diagnostic scenario we employ a LOOCV procedure whereby iteratively remove the cells from one patient sample from the dataset and train our CNN models on the cells from the rest of patient samples. After our models learn to distinguish between cells attained from patients in the training set, we then classify each individual cell of the “test” left-out patient sample. A majority voting is then applied whereby, the patient is classified to the category which is defined by the majority of its cellular predictions (see Supplementary Tables S1, S2). We find that training multiple binary classifiers boosts performance therefore, we revert to solving multiple 2 class problems to reach an optimal prediction accuracy (see Figure 3). We further split the data according to cell type, allowing for a prioritization of the different cell types available in the scRNA-seq data. The prioritization is obtained by ranking cell types according to the LOOCV AUC performance attained per cell type. We first apply our methodology on the MM dataset to specifically address the task of classifying early-stage MGUS and SMM patients (asymptomatic) vs. late-stage newly diagnosed NDMM patients (symptomatic). For this particular classification task, our best performing model was observed for the B-Cells, obtaining an AUC of 0.92, with high specificity (0.98) and a moderate sensitivity (0.57) (see Figure 4A). Aiming to boost our prediction accuracy even more, we proceeded to perform LOOCV experiments using mixtures of cell types. We used the top four scoring cell types from the single-type experiments (i.e., B-cells, erythroid-like and erythroid precursor cells, hematopoietic stem cells and platelets and performed LOOCV runs using all possible pairwise combinations of these. Result showed a substantial increase in model performance in accordance to our assessment metrics. Notably, we find that for the B-cells and erythroid-like and erythroid precursor cells mixture, the model sensitivity was significantly increased (0.86) as well specificity (1) and the overall AUC (0.97) (see Figure 4B).

**FIGURE 4:**
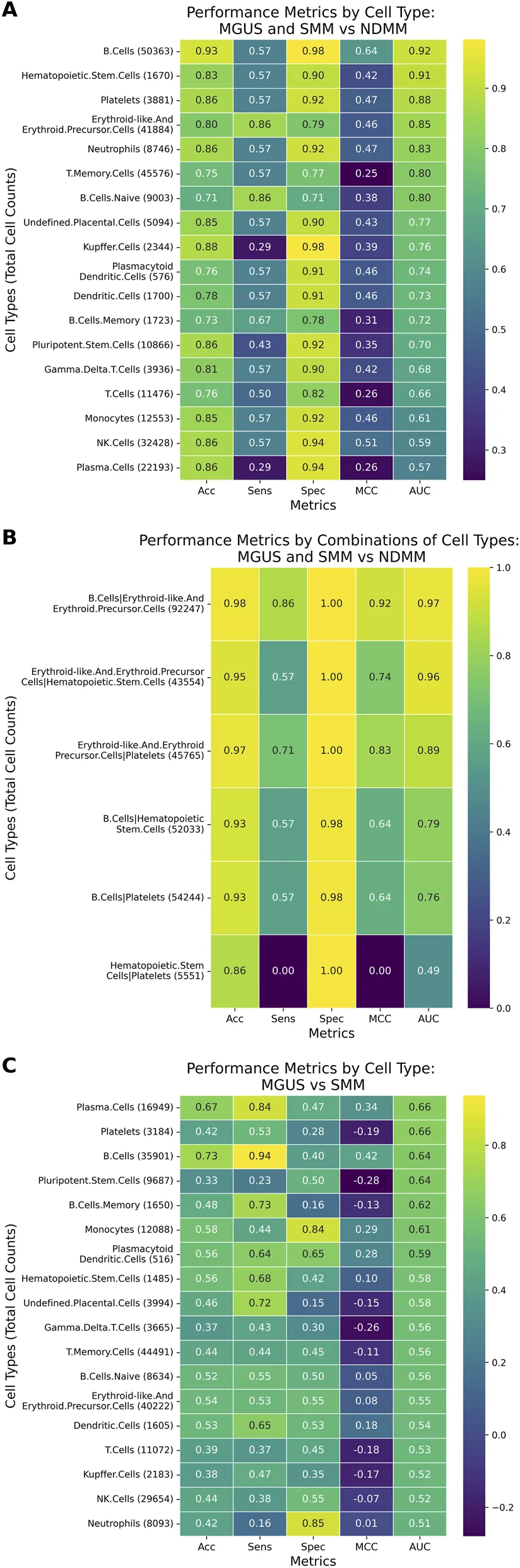
LOOCV for *Dang et al* MM dataset. **(A)** CNN models were trained to distinguish non-symptomatic diagnosed MM stages (MGUS and SMM) vs. pathological NDMM patients. B Cells and Hematopoietic stem cells are shown to obtain the highest LOOCV prediction accuracy for this particular binary classification problem. **(B)** Mixtures of Cell types further boost model performance for non-symptomatic (MGUS and SMM) vs. pathological (NDMM) stages of MM. B-cells and erythroid-like and erythroid precursor cells obtain optimal classification performance according to the metrics applied. **(C)** CNN models were trained to distinguish the two pro-symptomatic stages MGUS vs. SMM. Plasma Cells and platelets appear to obtain the highest LOOCV prediction accuracy for this particular binary classification problem. Acc = Prediction accuracy, Sens = Sensitivity, Pec = Specificity, MCC-Mathew’s Correlation, AUC = Area under the roc curve. Cell counts are also shown in the brackets next to the cell type names.

Next, we apply the same methodology to the task of separating the non-symptomatic early stages of MM (MGUS and SMM). These patients are often very difficult to classify clinically (Rajkumar et al., 2015) and this is also evident by the relatively low accuracy (AUC = 0.66) of our best prediction models (see Figure 4C).

In addition, results from our ablation study for this dataset show that when ANNs with the same architecture as DeepCellify were used, without incorporating IGTD into the classification process, model performance worsens. As outlined by our assessment metrics–best results obtained were for cell type hematopoietic stem cells, achieving: AUC = 0.95, sensitivity = 0.14 (very poor), specificity = 1 (see Supplementary Tables S3, S4). Interestingly, applying mixtures of cells in the ANNs used in the ablation study, again omitting the IGTD tabular-to-image conversion step, did not amount to the striking improvement in model performance as observed for mixed-cell samples with DeepCellify. Optimal results obtained were for mixtures of cell types hematopoietic stem cells and T cells, achieving: AUC = 0.97, sensitivity = 0.29, specificity = 1 (see Supplementary Table S5). Furthermore, we show that adopting other aggregation strategies such as probability voting and confidence-based voting slightly undervalue the performance of our models. However, lower confidence values were assigned to patient samples that were misclassified according to aggregation methods used (see Supplementary Table S6).

#### 3.1.2 COVID dataset

Next, we proceed to apply our training/validation and LOOCV methodology to the COVID dataset for the classification of COVID vs. non- COVID patients. Our best performing models (cell type, NK16.hi–see Figure 5), show a high training/validation AUC which is analogous to the AUC values reported by Mao et al. (2024) for the same dataset. In this recent publication the authors compare their tool ScRAT (Mao et al., 2024) with 5 other classification algorithms (attention-based, linear, feedforward (bulk), CloudPred (He et al., 2022), feedforward (single)) and report that ScRAT outperforms these tools with respect to validation performance across various training ratios. Thus, we continued to compare DeepCellify with the best ScRAT performance reported for the same dataset. Note, for comparison reasons we followed the same steps with the analysis process performed by Mao et al. (2024). Specifically, we performed 100 random train/validation splits (as performed by the authors in the ScRAT publications), and report our train/validation performance for our best models (see Supplementary Table S7). As by default DeepCellify calculates training performance at the cellular level, a shift to patient sample level was performed using majority voting from cellular predictions. These train/validation splits at different ratios, show that DeepCellify performs very well for this specific task, slightly outperforming previous ScRAT approaches (see Table 2). As an extension to this, we further proceed to analyse this dataset using a LOOCV procedure (not reported by *Mao et al* (Mao et al., 2024)) in order to simulate a diagnostic scenario in test patient samples. Under this scenario, we perform majority voting using cellular predictions (see Supplementary Table S8) and we also report a high classification accuracy (patient level), for our best performing models, that exceeds an AUC of 0.96, one again with balanced sensitivity and specificity values (see Figure 5, see Supplementary Figures S2, S3).

**FIGURE 5:**
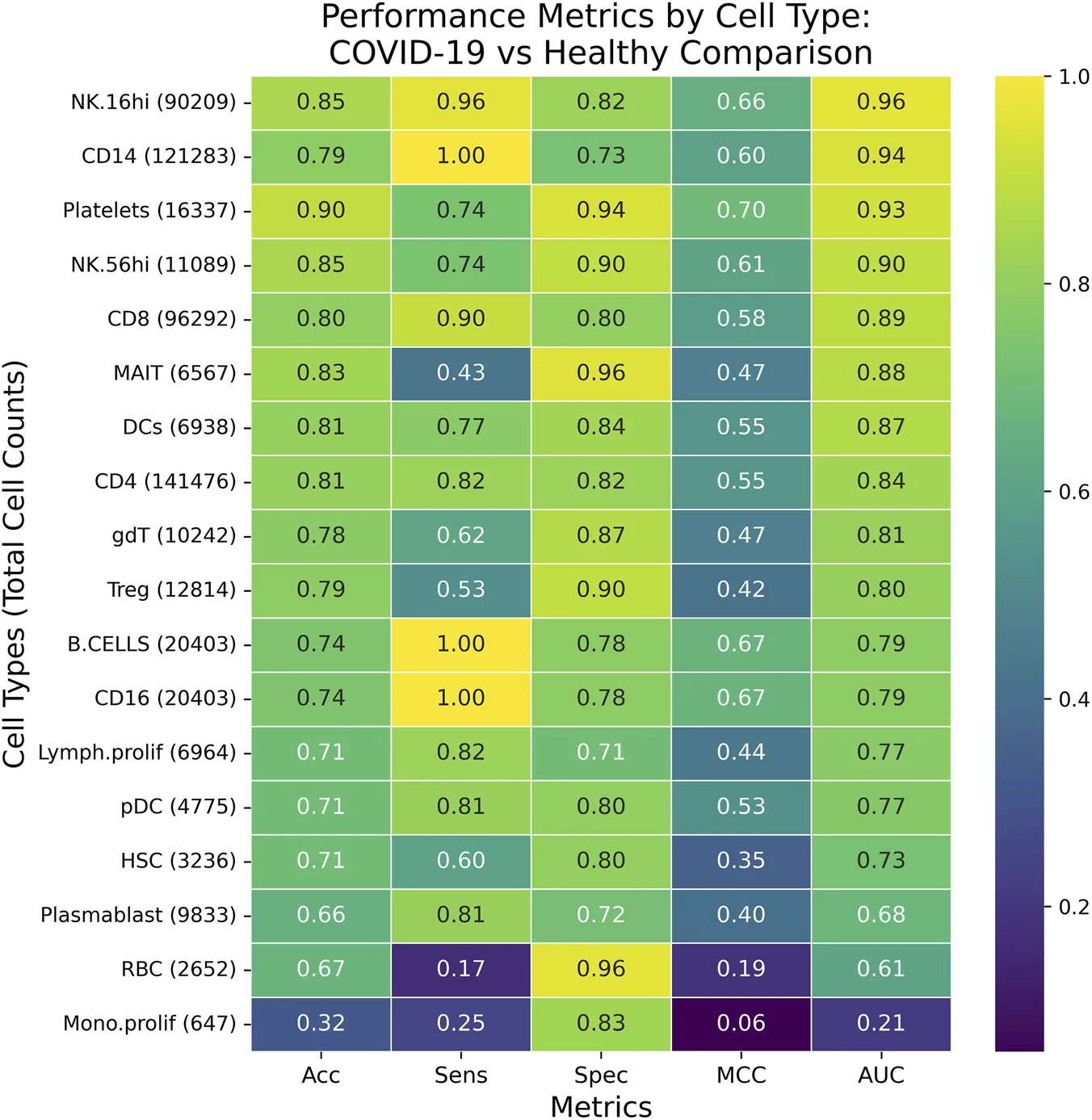
LOOCV for *Stephenson et al* COVID dataset. CNN models were trained to distinguish between disease (COVID) vs. healthy donors (non- COVID). NK and CD14 cells are shown to obtain the highest LOOCV prediction accuracy for this particular binary classification problem. Acc = Prediction accuracy, Sens = Sensitivity, Spec = Specificity, MCC-Mathew’s Correlation, AUC = Area under the roc curve. Cell counts are also shown in the brackets next to the cell type names.

**TABLE 2:** Comparison of DeepCellify with ScRAT on training/validation performance for the COVID dataset across different training ratios. For DeepCellify, averaging over 100 random splits is reported at the patient level.

Training Ratios	50%	80%
Number of Training Patient Samples	53	84
AUC		
ScRAT (PCA-b)	0.92	-
ScRAT (PCA-a)	0.92	-
DeepCellify	0.96	0.96
Precision		
ScRAT (PCA-b)	0.89	-
ScRAT (PCA-a)	0.88	-
DeepCellify	0.9	0.9
Recall/Sens		
ScRAT (PCA-b)	0.92	-
ScRAT (PCA-a)	0.92	-
DeepCellify	1	1

### 3.2 Analysis of differentially expressed genes (DEG) from DeepCellify classified cells generates biologically meaningful disease-related molecular signatures

Classifying individual cells constitutes one of DeepCellify’s unique characteristics. It allows for patient cells to be separated into those putatively exhibiting disease-related signals and those with limited evidence of such signals. We showcase this unique DeepCellify capability using the MM dataset as a case study. Specifically, we selected the MGUS/SMM vs. NDMM binary classification problem. We selected only the patient B-Cells which, were responsible for the best patient sample classification performance (see Figure 4A). After having classified all patient B-Cells into the two categories (MGUS/SMM vs. NDMM) during the majority voting step, we further proceeded to obtain molecular signatures for the two assigned cellular classes. Statistically significant genes upregulated in cells classified in accordance to the two MM dataset categories (MGUS/SMM and NDMM), revealed very interesting disease-related pathways after enrichment analysis, across four known pathway databases (see Figure 6).

**FIGURE 6:**
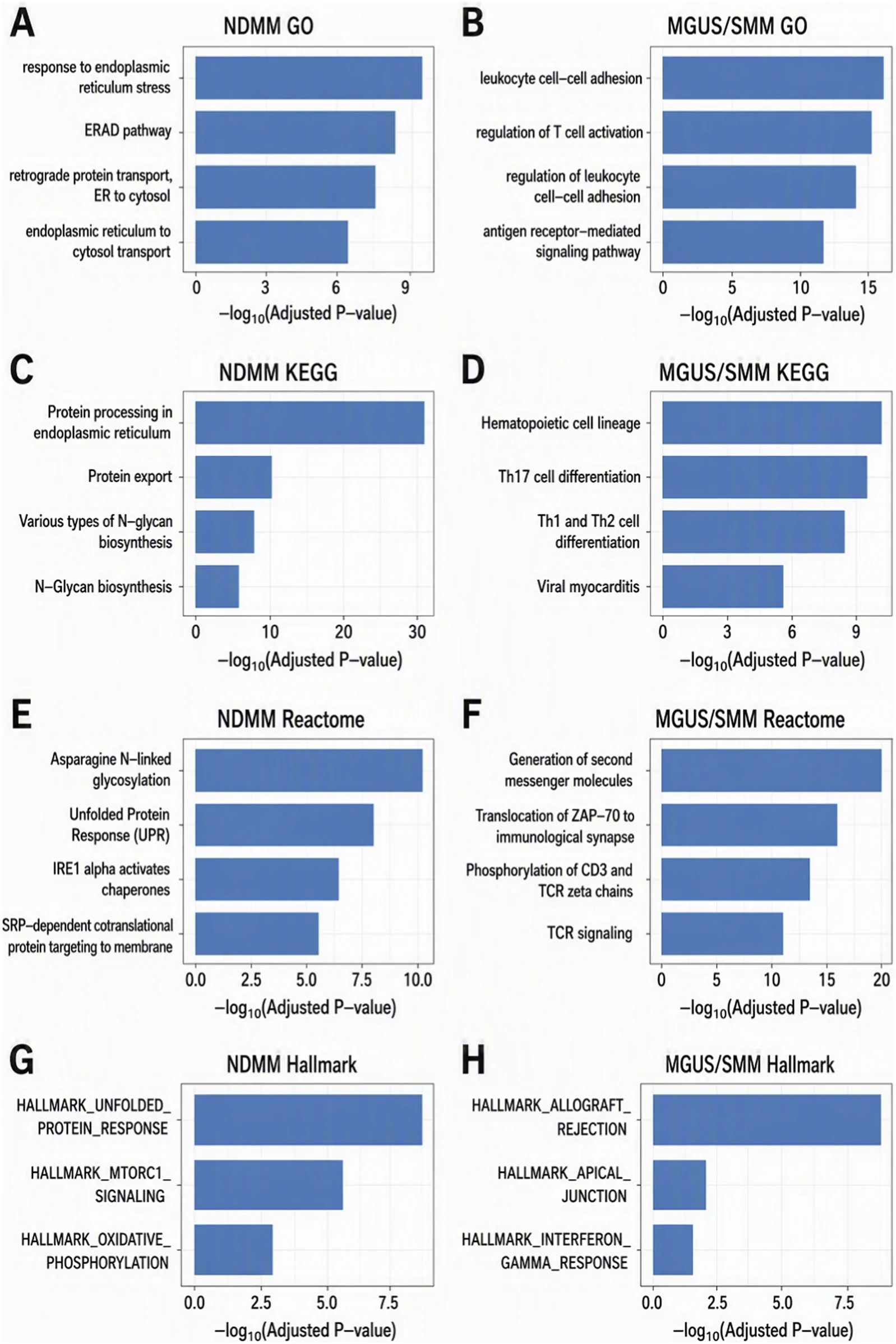
Functional pathway enrichment analysis of upregulated differentially expressed genes. Gene Ontology (GO), Kyoto Encyclopedia of Genes and Genomes (KEGG), Reactome, and Hallmark pathway enrichment analyses were performed for genes differentially expressed in NDMM and MGUS/SMM samples. The top significantly enriched pathways are shown for each database and disease group: **(A)** NDMM GO, **(B)** MGUS/SMM GO, **(C)** NDMM KEGG, **(D)** MGUS/SMM KEGG, **(E)** NDMM Reactome, **(F)** MGUS/SMM Reactome, **(G)** NDMM Hallmark, and **(H)** MGUS/SMM Hallmark. Bar lengths represent enrichment significance expressed as the negative logarithm of the adjusted P-value [−log10 (adjusted P-value)], with larger values indicating stronger statistical enrichment. Pathways associated with endoplasmic reticulum stress, protein processing and glycosylation predominated in NDMM, whereas immune regulation, T-cell activation, antigen presentation, and cell adhesion pathways were enriched in MGUS/SMM.

Genes upregulated in NDMM classified cells used as input to the GO database showed the following main enriched terms: response to ER stress, unfolded protein response, protein folding, ERAD pathway, protein glycosylation, N-linked glycosylation and protein targeting to ER. These pathways are directly relevant to MM and especially highly characteristic of symptomatic multiple myeloma. Specifically, myeloma plasma cells produce massive amounts of immunoglobulin, resulting in increased protein synthesis, heavy ER workload, dependence on unfolded protein response (UPR) signaling, increased glycosylation machinery and sensitivity to proteasome inhibition (Nikesitch et al., 2018; Borjan et al., 2020; Kubicki et al., 2021). These enrichment patterns directly reflect known vulnerabilities exploited by drugs such as bortezomib and carfilzomib (Borjan et al., 2020), thus alluding to an assessment that these predicted enriched pathways have a very strong myeloma-specific biological relevance.

Upregulated genes from the MGUS/SMM classified cells for the GO database showed very different pathway enriched terms. Namely: T-cell activation, T-cell receptor signaling, lymphocyte proliferation, antigen receptor signaling, leukocyte adhesion and immune activation. These terms show relevance to precursor MM specifically reflecting immune surveillance, anti-tumor immune responses and microenvironmental interactions. These are important during precursor stages, when immune control may still restrain plasma cell expansion, however, they are less specific to malignant plasma cells (McCachren et al., 2021). This suggests that these pathways are relevant to disease progression and immune context, but are less directly reflective of myeloma tumor biology.

KEGG Analysis using upregulated genes from NDMM cells revealed the following top pathways: protein processing in ER, protein export, N-glycan biosynthesis, oxidative phosphorylation and neurodegeneration pathways (Alzheimer, Parkinson, Huntington). Although neurodegeneration pathways seem unrelated to MM, they often appear because they share genes involved in mitochondrial metabolism, proteostasis and oxidative phosphorylation. The top three pathways are highly biologically meaningful as these are core features of antibody-secreting plasma cells (Nikesitch et al., 2018; Borjan et al., 2020; Kubicki et al., 2021). Thus, strongly linked to active myeloma biology.

Upregulated genes obtained from MGUS/SMM cells showed different themes according KEGG top pathways. These included: hematopoietic cell lineage, Th1/Th2 differentiation, Th17 differentiation, CAM interactions, primary immunodeficiency, allograft rejection and autoimmune disease pathways. These show high relevance to precursor MM. They indicate adaptive immune activation, immune-cell composition changes and microenvironment effects (McCachren et al., 2021). Useful for understanding precursor disease and immune regulation but less specific to malignant plasma cells.

Reactome Analysis for upregulated genes in the NDMM classified cells, showed the following top pathways: Asparagine N-linked glycosylation, unfolded protein response, IRE1α activates chaperones, XBP1 activates chaperones, ATF6 activates chaperones and protein targeting to membrane. Once again, the relevance to MM is striking as these are almost a textbook description of plasma-cell secretory biology. Particularly notable: XBP, IRE1α, ATF6. These are central UPR regulators known to support myeloma survival (Dong et al., 2019; Borjan et al., 2020). The enrichment strongly suggests dependence on ER stress adaptation, further alluding to signals that are extremely relevant to MM pathogenesis.

Upregulated genes for the MGUS/SMM cells showed different relevant enriched pathways in Reactome, with top pathways: TCR signaling, PD-1 co-inhibition, T-cell activation, antigen presentation, interferon-γ signaling and FCGR-mediated phagocytosis. These pathways are relevant to early stage MM as they indicate active immune surveillance, immune checkpoint regulation and antigen presentation (McCachren et al., 2021). Specifically, they are highly relevant to precursor disease and immunological control but not directly to plasma-cell oncogenic mechanisms.

Hallmark Analysis of upregulated genes from NDMM cells, showed the following enriched hallmark pathways: OXIDATIVE PHOSPHORYLATION, MTORC1 SIGNALING, UNFOLDED PROTEIN RESPONSE. All three are highly established features of myeloma such as increased metabolic demand, enhanced protein synthesis and dependence on ER stress management. Particularly important are UPR, mTOR activity and mitochondrial respiration (Zhan et al., 2017; Rizzieri et al., 2019; Weir et al., 2023). These support malignant plasma-cell growth, directly connected to MM pathophysiology.

Genes upregulated in MGUS/SMM cells again showed very different enriched Hallmark pathways show: INTERFERON GAMMA RESPONSE, ALLOGRAFT REJECTION and APICAL JUNCTION. These are again relevant to MM as they suggest immune activation, inflammatory responses and cell-cell interactions (McCachren et al., 2021). Themes which are albeit much less specific to malignant plasma cell and more relevant to precursor disease.

### 3.3 Mapping Grad-CAM activations to biologically informative genes for the MM dataset (“from pixels to genes”)

To enable biological interpretation of model predictions, Grad-CAM activation maps were systematically translated from image space back to gene-level features derived from the IGTD transformation. Grad-CAM highlights regions of the input image that contribute most strongly to the model’s prediction by leveraging gradients from the final convolutional layer. Given that IGTD embeds high-dimensional gene expression data into structured 2D images while preserving pairwise feature relationships, pixels in the Grad-CAM heatmap corresponds to specific gene locations in the original feature space.

Using the IGTD mapping, pixel-level importance scores were directly reassigned to their corresponding genes, enabling the identification of genes driving model decisions across samples. This approach provided a quantitative link between spatial activation patterns and underlying biological features, allowing the extraction of ranked gene importance profiles from model explanations.

Application of Grad-CAM to our trained CNN models with optimal performance for the MGUS|SMM vs. NDMM task (i.e., B-Cells models) produced localized heatmaps highlighting regions contributing most to classification decisions. These heatmaps were consistently sparse, with high-importance signals concentrated in specific pixel clusters rather than uniformly distributed across the image. This indicates that the model relies on a subset of informative genomic features rather than global patterns. Mapping Grad-CAM heatmaps back to gene space revealed a non-random distribution of feature importance. Across all samples, a subset of genes appeared repeatedly among the top-ranked contributors. Frequency analysis demonstrated that a small group of genes accounted for a disproportionately large number of high-importance activations, suggesting potential biological relevance.

Quantitatively, the top 5% of genes (40 genes) contributed to over 19.28% of cumulative Grad-CAM importance scores, indicating strong feature concentration (see Figure 7A).

**FIGURE 7:**
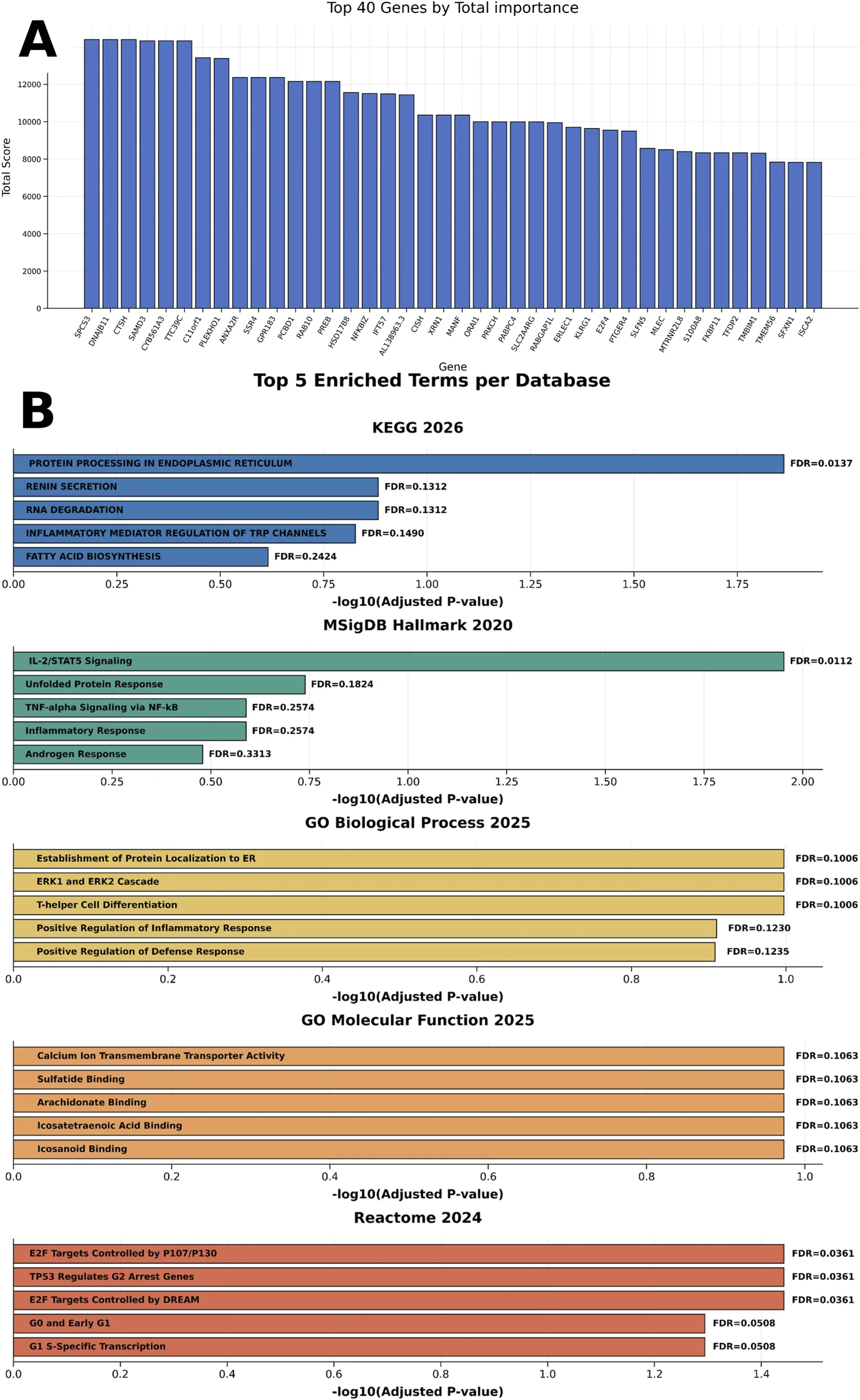
Grad-CAM interpretation results. **(A)** Top 40 genes mapped by Grad-CAM according to importance. **(B)** Functional pathway enrichment analysis for the Top 40 genes mapped by Grad-CAM. Prominent MM pathways are obsrved in accordance to previous results from the analysis of DEGs shown in Figure 6.

Gene set enrichment analysis, using enrichR (Xie et al., 2021), of the top 40 most important genes mapped by Grad-CAM revealed several genes mapped to well-established biological processes known to support MM pathophysiology (see Figure 7B; Supplementary Tables S9–S13). Multiple prominent MM themes were shared with the DEG analysis performed above using classified patient cells. The most prominent signal involved endoplasmic reticulum (ER) stress and the unfolded protein response (UPR), consistent with the high immunoglobulin secretory burden of malignant plasma cells. Genes including DNAJB11, MANF, ERLEC1, SSR4, FKBP11, and PREB are key components of protein folding, ER quality control, and secretory pathway regulation, all of which are essential for plasma cell survival and are known to be therapeutically targeted in MM via proteasome inhibition strategies (Xiong et al., 2021). A second major pathway involved NF-κB and cytokine signaling, represented by NFKBIZ, CISH, and PRKCH, which contribute to survival signaling, inflammatory regulation and drug resistance in MM cells. NF-κB activation is a well-established hallmark of MM pathogenesis and progression (Du et al., 2011; Sood et al., 2024). In addition, genes associated with calcium signaling and plasma cell function, including ORAI1, highlight dependence on store-operated calcium entry, which is critical for antibody secretion and plasma cell viability. Dysregulation of calcium homeostasis has been implicated in MM cell survival and stress adaptation (Li et al., 2021). Genes related to the immune microenvironment and cytotoxic response, such as PRF1, KLRG1, and S100A8, suggest alterations in immune surveillance and myeloid inflammation within the MM bone marrow niche. These findings align with evidence of T-cell dysfunction and inflammatory remodeling in MM (Kumar et al., 2017; Boiarsky et al., 2022). In addition, general cancer relevance genes were identified such as E2F4, a cell cycle transcription factor and of broad cancer relevance, including hematologic malignancies (Fulciniti et al., 2018), as well as TFDP2 which is a partner of E2F transcription factors.

For comparison reasons we also performed SHAP analysis in a similar manner to Grad-CAM. It was interesting to see that the two interpretation methods did not share top scoring genes. In addition, pathway enrichment analysis for SHAP (see Supplementary Tables S14–S19) did not concur with the prominent MM themes observed for Grad-CAM.

## 4 Discussion

The development of novel approaches for patient classification/stratification using high-throughput technologies is an ongoing field in bioinformatics. The advent of single-cell data allows for higher resolution and hence the potential to boost classification performance. Current approaches for the use of single-cell data to simulate scenarios of patient diagnostics are limited and often machine learning approaches report the training/validation performance of the trained model(s), without providing a real-time diagnostic solution using test data.

DeepCellify, provides a comprehensive analysis of scRNA-seq data specifically addressing the diagnostic potential of machine learning models. We developed a deep learning methodology based on CNNs, which utilizes a tabular to image conversion method that has not previously been employed for scRNA-seq data. We show that DeepCellify performs equally well and/or better to the state-of the art tools currently addressing patient classification using single-cell RNA-seq, and also offers some substantial advantages that entail: (i) Boosting the small number of patient samples (often less than 100); by allowing individual cells (often in the hundreds of thousands) to inherit the classification label of the patient from which they were derived from and consequently, using single cells as classification entities. (ii) Overcoming issues related to variability in cell numbers across different scRNA-seq samples. These may result in complications in utilizing accurate, representative expression matrices (genes vs. cells across samples) for classification. DeepCellify negates the need for such a matrix. (iii) Exploiting the fitting/training of CNN models using individual cell expression data to allow for the model to assign weights to the cells. Thus, allowing an automated cell weighting/ranking that selects for the “best” cells and cell types that are optimally suited for performing classification. Cells that have been incorrectly annotated or clustered may be repeatedly filtered out by the model. (iv) The application of our methodology in a diagnostic scenario by permitting trained CNN models to classify all cells obtained from a scRNA-seq experiment from an individual patient (test sample). A diagnosis is obtained by taking in consideration the class label that is given by our models to the majority of cells in the test sample(s).

In addition, in the MM case study, many of the cell types highlighted during the training/validation procedures are thoroughly studied in the context of MM (see Figure 4). For instance, in their article characterizing the role of the immune microenvironment in multiple myeloma progression at a single-cell level, Schinke et, al (Schinke et al., 2022) highlight plasma cells as the main driver of MM progression with their expansion correlating with disease severity. Other cell types such as B cells, T cells and natural killer (NK) cells are shown to play critical roles in shaping the immune microenvironment, with T and NK cells exhibiting reduced cytotoxic capacity, while B-cell compartments reflect broader immune dysregulation associated with disease progression (Zavidij et al., 2020; Schinke et al., 2022). Moreover, for the COVID-19 dataset, peripheral blood mononuclear cells (PBMCs) composition has previously been reported to vary depending on symptom duration, with increased relative frequency of plasmacytoid dendritic cells (pDCs), NK cells, CD14^+^ and CD16^+^ monocytes. Many of these cell types were also highlighted in our cell-type specific prioritization/ranking for this dataset (see Figure 5).

One of the unique characteristics of DeepCellify is its ability to classify cells into different signal-associated disease and/or control conditions. This permits analysis of DEGs between differentially classified cells. We thus apply a Wilcox test and further perform pathway enrichment analysis for highly statistically significant genes for one of the MM dataset binary problems, namely the MGUS/SMM vs. NDMM classification. Results show striking differences between pathways derived from cells classified in both disease conditions. Biological interpretation of the enrichments suggests a progression model for the early stage MGUS/SMM classified cells, characterized by a stronger immune-response signature, indicating active interaction between clonal plasma cells and the immune microenvironment. In contrast, the NDMM classified cells analysis transitions towards a secretory and metabolic plasma-cell theme, dominated by ER stress adaptation, UPR, glycosylation, protein export, and mitochondrial metabolism. Therefore, in all four databases (GO, KEGG, Reactome and Hallmark) the pathway enrichment analysis obtained from NDMM classified cells is more directly relevant to multiple myeloma because it captures the canonical plasma-cell dependencies on protein synthesis, unfolded protein response, ER stress management, glycosylation, and metabolic reprogramming that drive symptomatic disease. This type of cell classification into signal-associated disease and/or control conditions could be further strengthened by relevant studies on machine learning-based cell-type identification. A recently developed tool scMFF (Multiple Features Fusion Strategies for Cell Type Identification) (Sun et al., 2025), incorporates multiple complementary feature types including: statistical, information theory, matrix factorization, and deep learning. These representations are then modeled using multiple classifiers, such as SVM, LightGBM in order to predict cell-type. It would be interesting to investigate how such integrated multi-level approaches can perform on predicting putative cell conditions/states.

To bridge the gap between model interpretability and biological relevance, we translated Grad-CAM activation patterns from image space back to gene-level features using the IGTD-derived mapping. While Grad-CAM provides spatial explanations of model behavior, its direct interpretation is limited in the context of transformed tabular data. By leveraging the IGTD embedding, where gene expression features are arranged into a structured 2D representation preserving feature relationships, we were able to associate pixel-level importance with specific genes. This mapping framework enabled us to move beyond visual inspection of activation heatmaps and instead extract biologically interpretable signals driving model predictions. The approach highlights how deep learning models trained on image representations of omics data can still yield meaningful gene-level insights when appropriate reconstruction methods are applied. This was done using the MM models as a case study, and while no direct MM driver genes were identified, the genes mapped by Grad-CAM concurred with the previous DEG analysis performed using classified patient cells, strongly converging on ER stress adaptation, UPR, NF-κB signaling, calcium homeostasis, and immune microenvironment remodeling—core biological features of MM plasma cell survival.

When comparing Grad-CAM and SHAP results, we conclude that the interpretation provided by the former method is far more relevant to MM than the latter. Our contention is that Grad-CAM performed better than SHAP in our case because it captures high-level spatial features learned by the CNNs after integrating the four input channels, whereas SHAP produces noisy pixel-level attributions due to the strong spatial and channel correlations in the multi-channel cellular images.

Limitations of DeepCeelify, include long running times, which may affect scalability. In a single GPU NVIDIA, A30 set-up ∼19 h for a full dataset train/validation and LOOCV run (depending on the dataset). In addition, the process of reverting back to biologically informative genes from the cellular images is cumbersome and perhaps error prone. An important limitation of this approach is that the final feature–pixel permutation (index) generated by IGTD was not explicitly stored in the output files. Consequently, we approximated the mapping by inferring feature ordering from the IGTD “ranking_feature” matrix, which may not fully capture the exact optimized arrangement produced during training. This introduces a degree of uncertainty in the gene attribution process and should be considered when interpreting downstream biological findings. Future work should ensure that the exact index mapping is retained to enable precise and reproducible feature-level interpretations. Another limitation of this study is that the cell labels were derived using a weakly supervised approach rather than independent experimental validation. Therefore, we interpret the assigned cell groups as reflecting inferred or putative disease-signal associations rather than definitive biological states.

In conclusion, the tabular to image converter tool (IGTD) transformation successfully converted high-dimensional tabular scRNA gene expression data into structured 2D image representations (30 × 30 pixels), preserving feature similarity relationships. Each pixel corresponded to a mapped gene, enabling direct interpretability of convolutional neural network (CNN) outputs. The IGTD tool, used herein, has previously been applied for the conversion of bulk-RNA gene expression data to an image and examples of classification of patients based on this conversion has been documented (Zhu et al., 2021). However, to the best of our knowledge, its application within a single-cell framework has not previously been reported; here, we provide evidence supporting its novel use in this context.

Finally, given the high accuracy of our classification models and the relevance of the cell-types, as well as biologically informative genes, highlighted by our patient stratification schemes, we propose that our methodology can potentially be used in a clinical set-up providing useful support to clinicians, when scRNA-seq data is made available. This is not something which may currently be in practice, due to the cost and complexity of scRNA-seq data, hence, hindering their applicability in diagnostics. However, the enhanced resolution offered by scRNA-seq data is likely to be critical for the development of more advanced diagnostic strategies. Furthermore, the insights derived from our methodology may be transferable to more accessible platforms, such as flow cytometry, enabling the potential adaptation of the classification framework to clinically established assays and thereby enhancing its practical applicability.

The nature of the conversion of the tabular expression cellular expression data to an image adopted by DeepCellify for scRNA-seq data, permits the direct comparison of distance matrices (similarities), obtained by intra-sample calculations, after rendering them in the form of images, while remaining independent of dataset specific variation. Future prospects include investigating whether our approach can potentially allow the assessment of scRNA-seq samples across different datasets without the need for cumbersome batch correction methods that often lead to erroneous results.

## Data Availability

Publicly available datasets were analyzed in this study. This data can be found here: The Multiple Myeloma (MM) dataset is available for download from the European Genome-phenome Archive (EGA) using the database ID: EGAD00001009648. The COVID-19 dataset is available for download from the authors weblink, as a h5ad file, from https://covid19cellatlas.org/. The processed COVID-19 data are available to download from Array Express under accession number E-MTAB-10026.
