# Supplementary material for: Patient classification using single-cell RNA-sequencing data with deep learning convolutional neural networks
Source: Front Bioinform. 2026 Sep 29;6:1919127. doi: 10.3389/fbinf.2026.1919127 (PMC13658557; doi:10.3389/fbinf.2026.1919127)
Supplement: Supplementary file 1 [file Supplementaryfile1.docx]

**Supplementary Material**

**Patient Classification using single-cell RNA-Sequencing data with Deep Learning Convolutional Neural Networks**

Figure S1. Typical image generated by IGDT from tabular scRNA-Seq data. Image represents a single cell from the Multiple Myeloma (MM) Dataset (B-Cell Naive cell, from MGUS01s patient sample).

Figure S2. Typical metrics monitoring across epochs during validation runs at the 50% training ratio. Early stoppage is viewed due no change in validation AUC (val_auc) for 10 consecutive epochs or min delta = 0.01.

Figure S3. Typical ROC curve from single validation run at the 50% ratio

**CNN** **Hyperparameter tuning and model selection.**

To identify the optimal convolutional neural network (CNN) architecture, we performed systematic hyperparameter tuning by evaluating multiple combinations of network depth, convolutional filter numbers, kernel sizes, stride lengths, pooling strategies, dropout rates, and dense layer dimensions. Candidate models were trained and assessed using cross-validation on the training dataset, with model selection based primarily on validation performance (AUC) while minimizing overfitting. Architectures ranging from two to four convolutional layers, filter sizes of 32-128, dropout rates between 0.1 and 0.5, and dense layer sizes between 25 and 200 neurons were explored. The final architecture demonstrated the best balance between predictive performance and generalization and consisted of four convolutional layers with 32, 32, 64, and 64 filters (3 × 3 kernels), interleaved with max-pooling and dropout layers (rate = 0.25), followed by a fully connected layer containing 50 neurons and a sigmoid-activated output layer for binary classification. ReLU activation functions were employed throughout the hidden layers.

Table S1. LOOCV Majority voting for MGUS|SMM vs NDMM classification. All patient samples are assigned to a specific class based on the majority of their cellular predictions.

| Sample ID_Class_CellType | MGUS\|SMM | NDMM | Majority_Voting |
| --- | --- | --- | --- |
| MGUS01s_B.Cells | 460 | 47 | MGUS_SMM |
| MGUS02s_B.Cells | 881 | 1 | MGUS_SMM |
| MGUS03s_B.Cells | 121 | 10 | MGUS_SMM |
| MGUS04s_B.Cells | 106 | 19 | MGUS_SMM |
| MGUS05s_B.Cells | 3450 | 1425 | MGUS_SMM |
| MGUS06s_B.Cells | 863 | 2100 | NDMM |
| MGUS07_B.Cells | 73 | 2 | MGUS_SMM |
| MGUS08_B.Cells | 765 | 13 | MGUS_SMM |
| MGUS09_B.Cells | 1046 | 3 | MGUS_SMM |
| MGUS10_B.Cells | 571 | 0 | MGUS_SMM |
| MGUS11_B.Cells | 125 | 6 | MGUS_SMM |
| MGUS12_B.Cells | 11 | 0 | MGUS_SMM |
| MGUS13_B.Cells | 884 | 2 | MGUS_SMM |
| MGUS14_B.Cells | 276 | 2 | MGUS_SMM |
| MGUS16_B.Cells | 240 | 1 | MGUS_SMM |
| MGUS17_B.Cells | 222 | 2 | MGUS_SMM |
| MGUS18_B.Cells | 1759 | 133 | MGUS_SMM |
| MGUS19_B.Cells | 151 | 8 | MGUS_SMM |
| MGUS20_B.Cells | 1206 | 5 | MGUS_SMM |
| MGUS21_B.Cells | 566 | 4 | MGUS_SMM |
| NDMM01s_B.Cells | 2836 | 239 | MGUS_SMM |
| NDMM02s_B.Cells | 63 | 1818 | NDMM |
| NDMM03s_B.Cells | 410 | 1977 | NDMM |
| NDMM04s_B.Cells | 83 | 1307 | NDMM |
| NDMM05s_B.Cells | 182 | 5010 | NDMM |
| NDMM06_B.Cells | 259 | 88 | MGUS_SMM |
| NDMM07_B.Cells | 172 | 18 | MGUS_SMM |
| SMM01s_B.Cells | 2113 | 643 | MGUS_SMM |
| SMM02s_B.Cells | 280 | 11 | MGUS_SMM |
| SMM03s_B.Cells | 699 | 72 | MGUS_SMM |
| SMM04s_B.Cells | 1082 | 314 | MGUS_SMM |
| SMM05s_B.Cells | 707 | 550 | MGUS_SMM |
| SMM06s_B.Cells | 30 | 12 | MGUS_SMM |
| SMM07s_B.Cells | 123 | 74 | MGUS_SMM |
| SMM08_B.Cells | 97 | 3 | MGUS_SMM |
| SMM10_B.Cells | 54 | 2 | MGUS_SMM |
| SMM11_B.Cells | 29 | 4 | MGUS_SMM |
| SMM12_B.Cells | 138 | 9 | MGUS_SMM |
| SMM13_B.Cells | 132 | 3 | MGUS_SMM |
| SMM14_B.Cells | 140 | 0 | MGUS_SMM |
| SMM15_B.Cells | 745 | 3 | MGUS_SMM |
| SMM16_B.Cells | 525 | 2 | MGUS_SMM |
| SMM17_B.Cells | 1713 | 4 | MGUS_SMM |
| SMM18_B.Cells | 876 | 20 | MGUS_SMM |
| SMM19_B.Cells | 661 | 1 | MGUS_SMM |
| SMM20_B.Cells | 549 | 7 | MGUS_SMM |
| SMM21_B.Cells | 282 | 30 | MGUS_SMM |
| SMM22_B.Cells | 169 | 3 | MGUS_SMM |
| SMM23_B.Cells | 370 | 5 | MGUS_SMM |
| SMM24_B.Cells | 225 | 11 | MGUS_SMM |
| SMM25_B.Cells | 949 | 7 | MGUS_SMM |
| SMM26_B.Cells | 163 | 23 | MGUS_SMM |
| SMM27_B.Cells | 752 | 15 | MGUS_SMM |
| SMM28_B.Cells | 462 | 42 | MGUS_SMM |
| SMM29_B.Cells | 281 | 2 | MGUS_SMM |
| SMM30_B.Cells | 528 | 7 | MGUS_SMM |
| SMM31_B.Cells | 665 | 8 | MGUS_SMM |
| SMM32_B.Cells | 165 | 1 | MGUS_SMM |
| SMM33_B.Cells | 742 | 8 | MGUS_SMM |

Table S2. LOOCV Majority voting for MGUS vs SMM classification. All patient samples are assigned to a specific class based on the majority of their cellular predictions.

| Sample ID_Class_CellType | MGUS | SMM | Majority_Voting |
| --- | --- | --- | --- |
| MGUS01s_Plasma.Cells | 103 | 350 | SMM |
| MGUS02s_Plasma.Cells | 18 | 224 | SMM |
| MGUS03s_Plasma.Cells | 141 | 105 | MGUS |
| MGUS04s_Plasma.Cells | 80 | 71 | MGUS |
| MGUS05s_Plasma.Cells | 140 | 1422 | SMM |
| MGUS06s_Plasma.Cells | 249 | 1766 | SMM |
| MGUS07_Plasma.Cells | 9 | 47 | SMM |
| MGUS08_Plasma.Cells | 36 | 37 | SMM |
| MGUS09_Plasma.Cells | 48 | 80 | SMM |
| MGUS10_Plasma.Cells | 49 | 44 | MGUS |
| MGUS11_Plasma.Cells | 36 | 69 | SMM |
| MGUS12_Plasma.Cells | 1 | 0 | MGUS |
| MGUS13_Plasma.Cells | 45 | 28 | MGUS |
| MGUS14_Plasma.Cells | 18 | 13 | MGUS |
| MGUS16_Plasma.Cells | 13 | 14 | SMM |
| MGUS17_Plasma.Cells | 24 | 26 | SMM |
| MGUS18_Plasma.Cells | 596 | 421 | MGUS |
| MGUS19_Plasma.Cells | 5 | 5 | MGUS |
| MGUS20_Plasma.Cells | 44 | 43 | MGUS |
| MGUS21_Plasma.Cells | 46 | 22 | MGUS |
| SMM01s_Plasma.Cells | 175 | 998 | SMM |
| SMM02s_Plasma.Cells | 223 | 98 | MGUS |
| SMM03s_Plasma.Cells | 137 | 538 | SMM |
| SMM04s_Plasma.Cells | 725 | 784 | SMM |
| SMM05s_Plasma.Cells | 18 | 678 | SMM |
| SMM06s_Plasma.Cells | 11 | 21 | SMM |
| SMM07s_Plasma.Cells | 28 | 200 | SMM |
| SMM08_Plasma.Cells | 3 | 192 | SMM |
| SMM10_Plasma.Cells | 2 | 6 | SMM |
| SMM11_Plasma.Cells | 6 | 121 | SMM |
| SMM12_Plasma.Cells | 68 | 241 | SMM |
| SMM13_Plasma.Cells | 17 | 64 | SMM |
| SMM14_Plasma.Cells | 1 | 1 | MGUS |
| SMM15_Plasma.Cells | 226 | 1 | MGUS |
| SMM16_Plasma.Cells | 21 | 85 | SMM |
| SMM17_Plasma.Cells | 48 | 119 | SMM |
| SMM18_Plasma.Cells | 144 | 147 | SMM |
| SMM19_Plasma.Cells | 6 | 57 | SMM |
| SMM20_Plasma.Cells | 62 | 160 | SMM |
| SMM21_Plasma.Cells | 120 | 339 | SMM |
| SMM22_Plasma.Cells | 46 | 348 | SMM |
| SMM23_Plasma.Cells | 71 | 180 | SMM |
| SMM24_Plasma.Cells | 28 | 77 | SMM |
| SMM25_Plasma.Cells | 222 | 130 | MGUS |
| SMM26_Plasma.Cells | 26 | 393 | SMM |
| SMM27_Plasma.Cells | 14 | 62 | SMM |
| SMM28_Plasma.Cells | 60 | 1219 | SMM |
| SMM29_Plasma.Cells | 126 | 76 | MGUS |
| SMM30_Plasma.Cells | 63 | 73 | SMM |
| SMM31_Plasma.Cells | 11 | 16 | SMM |
| SMM32_Plasma.Cells | 20 | 17 | MGUS |
| SMM33_Plasma.Cells | 84 | 208 | SMM |

Table S3. Neural Network ablation study (excluding IGTD) results for MGUS|SMM vs NDMM classification.

| cell_type | auc_patient | sensitivity_patient | specificity_patient | accuracy_patient |
| --- | --- | --- | --- | --- |
| Hematopoietic.Stem.Cells | 0.95 | 0.14 | 1.00 | 0.89 |
| T.Memory.Cells | 0.94 | 0.14 | 1.00 | 0.90 |
| Pluripotent.Stem.Cells | 0.93 | 0.14 | 1.00 | 0.90 |
| T.Cells | 0.91 | 0.29 | 1.00 | 0.91 |
| Kupffer.Cells | 0.90 | 0.14 | 1.00 | 0.89 |
| Plasmacytoid.Dendritic.Cells | 0.90 | 0.14 | 0.98 | 0.87 |
| Neutrophils | 0.89 | 0.14 | 1.00 | 0.90 |
| Platelets | 0.88 | 0.57 | 0.98 | 0.93 |
| B.Cells.Naive | 0.87 | 0.43 | 0.98 | 0.91 |
| B.Cells | 0.86 | 0.14 | 1.00 | 0.90 |
| NK.Cells | 0.85 | 0.14 | 1.00 | 0.90 |
| Erythroid-like.And.Erythroid.Precursor.Cells | 0.79 | 0.14 | 0.96 | 0.86 |
| Monocytes | 0.79 | 0.14 | 1.00 | 0.90 |
| Undefined.Placental.Cells | 0.78 | 0.14 | 0.96 | 0.86 |
| Gamma.Delta.T.Cells | 0.78 | 0.43 | 0.94 | 0.87 |
| Plasma.Cells | 0.69 | 0.14 | 1.00 | 0.90 |
| Dendritic.Cells | 0.62 | 0.29 | 0.98 | 0.88 |
| B.Cells.Memory | 0.61 | 0.00 | 0.96 | 0.85 |

Table S4. Neural Network ablation study (excluding IGTD) results for MGUS vs SMM classification.

| cell_type | auc_patient | sensitivity_patient | specificity_patient | accuracy_patient |
| --- | --- | --- | --- | --- |
| Pluripotent.Stem.Cells | 0.68 | 0.13 | 0.84 | 0.39 |
| B.Cells | 0.67 | 0.16 | 0.85 | 0.42 |
| Platelets | 0.67 | 0.52 | 0.68 | 0.58 |
| Gamma.Delta.T.Cells | 0.65 | 0.14 | 0.89 | 0.43 |
| Plasma.Cells | 0.63 | 0.88 | 0.05 | 0.57 |
| Erythroid-like.And.Erythroid.Precursor.Cells | 0.62 | 0.16 | 0.90 | 0.44 |
| Monocytes | 0.62 | 0.19 | 0.84 | 0.43 |
| NK.Cells | 0.58 | 0.25 | 0.85 | 0.48 |
| T.Cells | 0.57 | 0.13 | 0.84 | 0.39 |
| Neutrophils | 0.56 | 0.16 | 0.90 | 0.44 |
| B.Cells.Naive | 0.54 | 0.17 | 0.84 | 0.43 |
| Hematopoietic.Stem.Cells | 0.52 | 0.21 | 0.89 | 0.48 |
| Kupffer.Cells | 0.51 | 0.16 | 0.89 | 0.44 |
| Undefined.Placental.Cells | 0.50 | 0.23 | 0.68 | 0.40 |
| Plasmacytoid.Dendritic.Cells | 0.50 | 0.24 | 0.75 | 0.42 |
| Dendritic.Cells | 0.50 | 0.31 | 0.71 | 0.47 |
| B.Cells.Memory | 0.49 | 0.18 | 0.79 | 0.43 |
| T.Memory.Cells | 0.46 | 0.69 | 0.35 | 0.56 |

Table S5. Neural Network ablation study (excluding IGTD) results for mixed cell types for MGUS|SMM vs NDMM classification.

| cell_type | auc_patient | sensitivity_patient | specificity_patient | accuracy_patient |
| --- | --- | --- | --- | --- |
| Hematopoietic.Stem.Cells_T.Cells | 0.97 | 0.29 | 1.00 | 0.92 |
| Hematopoietic.Stem.Cells_Pluripotent.Stem.Cells_T.Cells | 0.96 | 0.14 | 1.00 | 0.90 |
| Hematopoietic.Stem.Cells_Pluripotent.Stem.Cells | 0.94 | 0.14 | 1.00 | 0.90 |
| Pluripotent.Stem.Cells_T.Cells | 0.94 | 0.29 | 0.98 | 0.90 |
| T.Memory.Cells_Pluripotent.Stem.Cells | 0.93 | 0.29 | 1.00 | 0.92 |
| T.Memory.Cells_Pluripotent.Stem.Cells_T.Cells | 0.93 | 0.29 | 1.00 | 0.92 |
| Hematopoietic.Stem.Cells_T.Memory.Cells_Pluripotent.Stem.Cells | 0.93 | 0.29 | 0.98 | 0.90 |
| T.Memory.Cells_T.Cells | 0.93 | 0.29 | 1.00 | 0.92 |
| Hematopoietic.Stem.Cells_T.Memory.Cells | 0.91 | 0.29 | 0.98 | 0.90 |
| Hematopoietic.Stem.Cells_T.Memory.Cells_T.Cells | 0.91 | 0.29 | 1.00 | 0.92 |
| B.Cells_Erythroid-like.And.Erythroid.Precursor.Cells | 0.82 | 0.14 | 1.00 | 0.90 |

Table S6. Probability voting and Confidence based voting results show sensitivity = 0.5714286, specificity = 0.9615385, accuracy = 0.9152542, AUC= 0.7335. Results from majority voting are overall better according to metrics used to assess performance.

| Sample | Probability | Confidence | Prediction |
| --- | --- | --- | --- |
| MGUS01s_B.Cells | 0.06025 | 0.43975 | MGUS\|SMM |
| MGUS02s_B.Cells | 0.016745 | 0.483255 | MGUS\|SMM |
| MGUS03s_B.Cells | 0.049944 | 0.450056 | MGUS\|SMM |
| MGUS04s_B.Cells | 0.138553 | 0.361447 | MGUS\|SMM |
| MGUS05s_B.Cells | 0.577478 | 0.077478 | NDMM |
| MGUS06s_B.Cells | 0.656511 | 0.156511 | NDMM |
| MGUS07_B.Cells | 0.03414 | 0.46586 | MGUS\|SMM |
| MGUS08_B.Cells | 0.020448 | 0.479552 | MGUS\|SMM |
| MGUS09_B.Cells | 0.02245 | 0.47755 | MGUS\|SMM |
| MGUS10_B.Cells | 0.007381 | 0.492619 | MGUS\|SMM |
| MGUS11_B.Cells | 0.061331 | 0.438669 | MGUS\|SMM |
| MGUS12_B.Cells | 0.003689 | 0.496311 | MGUS\|SMM |
| MGUS13_B.Cells | 0.019358 | 0.480642 | MGUS\|SMM |
| MGUS14_B.Cells | 0.019089 | 0.480911 | MGUS\|SMM |
| MGUS16_B.Cells | 0.024523 | 0.475477 | MGUS\|SMM |
| MGUS17_B.Cells | 0.031885 | 0.468115 | MGUS\|SMM |
| MGUS18_B.Cells | 0.146283 | 0.353717 | MGUS\|SMM |
| MGUS19_B.Cells | 0.066297 | 0.433703 | MGUS\|SMM |
| MGUS20_B.Cells | 0.016899 | 0.483101 | MGUS\|SMM |
| MGUS21_B.Cells | 0.014808 | 0.485192 | MGUS\|SMM |
| NDMM01s_B.Cells | 0.006764 | 0.493236 | MGUS\|SMM |
| NDMM02s_B.Cells | 0.925614 | 0.425614 | NDMM |
| NDMM03s_B.Cells | 0.620016 | 0.120016 | NDMM |
| NDMM04s_B.Cells | 0.776047 | 0.276047 | NDMM |
| NDMM05s_B.Cells | 0.916379 | 0.416379 | NDMM |
| NDMM06_B.Cells | 0.034139 | 0.465861 | MGUS\|SMM |
| NDMM07_B.Cells | 0.063334 | 0.436666 | MGUS\|SMM |
| SMM01s_B.Cells | 0.239268 | 0.260732 | MGUS\|SMM |
| SMM02s_B.Cells | 0.034247 | 0.465753 | MGUS\|SMM |
| SMM03s_B.Cells | 0.213243 | 0.286757 | MGUS\|SMM |
| SMM04s_B.Cells | 0.287491 | 0.212509 | MGUS\|SMM |
| SMM05s_B.Cells | 0.457028 | 0.042972 | MGUS\|SMM |
| SMM06s_B.Cells | 0.381578 | 0.118422 | MGUS\|SMM |
| SMM07s_B.Cells | 0.203876 | 0.296124 | MGUS\|SMM |
| SMM08_B.Cells | 0.065205 | 0.434795 | MGUS\|SMM |
| SMM10_B.Cells | 0.054947 | 0.445053 | MGUS\|SMM |
| SMM11_B.Cells | 0.235916 | 0.264084 | MGUS\|SMM |
| SMM12_B.Cells | 0.090472 | 0.409528 | MGUS\|SMM |
| SMM13_B.Cells | 0.017222 | 0.482778 | MGUS\|SMM |
| SMM14_B.Cells | 0.019737 | 0.480263 | MGUS\|SMM |
| SMM15_B.Cells | 0.019897 | 0.480103 | MGUS\|SMM |
| SMM16_B.Cells | 0.01611 | 0.48389 | MGUS\|SMM |
| SMM17_B.Cells | 0.014481 | 0.485519 | MGUS\|SMM |
| SMM18_B.Cells | 0.062446 | 0.437554 | MGUS\|SMM |
| SMM19_B.Cells | 0.00807 | 0.49193 | MGUS\|SMM |
| SMM20_B.Cells | 0.016942 | 0.483058 | MGUS\|SMM |
| SMM21_B.Cells | 0.093004 | 0.406996 | MGUS\|SMM |
| SMM22_B.Cells | 0.069698 | 0.430302 | MGUS\|SMM |
| SMM23_B.Cells | 0.017405 | 0.482595 | MGUS\|SMM |
| SMM24_B.Cells | 0.058221 | 0.441779 | MGUS\|SMM |
| SMM25_B.Cells | 0.016307 | 0.483693 | MGUS\|SMM |
| SMM26_B.Cells | 0.210485 | 0.289515 | MGUS\|SMM |
| SMM27_B.Cells | 0.016479 | 0.483521 | MGUS\|SMM |
| SMM28_B.Cells | 0.107132 | 0.392868 | MGUS\|SMM |
| SMM29_B.Cells | 0.022593 | 0.477407 | MGUS\|SMM |
| SMM30_B.Cells | 0.022402 | 0.477598 | MGUS\|SMM |
| SMM31_B.Cells | 0.019097 | 0.480903 | MGUS\|SMM |
| SMM32_B.Cells | 0.02951 | 0.47049 | MGUS\|SMM |
| SMM33_B.Cells | 0.040459 | 0.459541 | MGUS\|SMM |

Table S7. 100 random training validations splits (50% training ratio) for COVID vs Healthy classification. Predictions were converted from the cellular (DeepCellify default) to the patient level by averaging model-derived probabilities across cells per patient sample. Threshold were calculated according to Youden's J statistic:

| Training Run | Optimal Youden’s threshold | precision | recall | specificity |
| --- | --- | --- | --- | --- |
| 0 | 0.42 | 0.851852 | 1 | 0.955056 |
| 1 | 0.46 | 0.884615 | 1 | 0.966292 |
| 2 | 0.47 | 0.958333 | 1 | 0.988764 |
| 3 | 0.43 | 0.884615 | 1 | 0.966292 |
| 4 | 0.52 | 0.884615 | 1 | 0.966292 |
| 5 | 0.48 | 0.884615 | 1 | 0.966292 |
| 6 | 0.44 | 0.92 | 1 | 0.977528 |
| 7 | 0.53 | 0.92 | 1 | 0.977528 |
| 8 | 0.49 | 0.92 | 1 | 0.977528 |
| 9 | 0.49 | 0.92 | 1 | 0.977528 |
| 10 | 0.53 | 1 | 0.956522 | 1 |
| 11 | 0.43 | 0.884615 | 1 | 0.966292 |
| 12 | 0.46 | 0.884615 | 1 | 0.966292 |
| 13 | 0.55 | 0.92 | 1 | 0.977528 |
| 14 | 0.47 | 0.884615 | 1 | 0.966292 |
| 15 | 0.51 | 0.92 | 1 | 0.977528 |
| 16 | 0.49 | 0.92 | 1 | 0.977528 |
| 17 | 0.42 | 0.851852 | 1 | 0.955056 |
| 18 | 0.53 | 0.884615 | 1 | 0.966292 |
| 19 | 0.46 | 0.851852 | 1 | 0.955056 |
| 20 | 0.49 | 0.884615 | 1 | 0.966292 |
| 21 | 0.45 | 0.851852 | 1 | 0.955056 |
| 22 | 0.52 | 0.92 | 1 | 0.977528 |
| 23 | 0.42 | 0.92 | 1 | 0.977528 |
| 24 | 0.46 | 0.884615 | 1 | 0.966292 |
| 25 | 0.49 | 0.884615 | 1 | 0.966292 |
| 26 | 0.42 | 0.884615 | 1 | 0.966292 |
| 27 | 0.43 | 0.92 | 1 | 0.977528 |
| 28 | 0.45 | 0.92 | 1 | 0.977528 |
| 29 | 0.38 | 0.821429 | 1 | 0.94382 |
| 30 | 0.51 | 0.884615 | 1 | 0.966292 |
| 31 | 0.37 | 0.92 | 1 | 0.977528 |
| 32 | 0.5 | 0.851852 | 1 | 0.955056 |
| 33 | 0.45 | 0.884615 | 1 | 0.966292 |
| … | … | … | … | … |
| 100 | … | … | … | … |
| Average |  | 0.896 | 0.9987 | 0.9696 |

Table S8. LOOCV Majority voting for COVID vs Healthy classification. All patient samples are assigned to a specific class based on the majority of their cellular predictions.

| Sample ID_Class_CellType | Covid | Healthy | Majority_Voting |
| --- | --- | --- | --- |
| AP1_Covid_NK.16hi | 376 | 68 | Covid |
| AP10_Covid_NK.16hi | 186 | 4 | Covid |
| AP11_Covid_NK.16hi | 599 | 15 | Covid |
| AP12_Covid_NK.16hi | 2035 | 119 | Covid |
| AP2_Covid_NK.16hi | 76 | 29 | Covid |
| AP3_Covid_NK.16hi | 243 | 1 | Covid |
| AP4_Covid_NK.16hi | 150 | 293 | Healthy |
| AP5_Covid_NK.16hi | 1217 | 67 | Covid |
| AP6_Covid_NK.16hi | 3866 | 168 | Covid |
| AP8_Covid_NK.16hi | 262 | 13 | Covid |
| AP9_Covid_NK.16hi | 261 | 90 | Covid |
| CV0025_Covid_NK.16hi | 141 | 45 | Covid |
| CV0037_Covid_NK.16hi | 123 | 27 | Covid |
| CV0050_Covid_NK.16hi | 139 | 246 | Healthy |
| CV0052_Covid_NK.16hi | 183 | 22 | Covid |
| CV0058_Covid_NK.16hi | 87 | 25 | Covid |
| CV0059_Covid_NK.16hi | 380 | 543 | Healthy |
| CV0062_Covid_NK.16hi | 189 | 275 | Healthy |
| CV0068_Covid_NK.16hi | 101 | 316 | Healthy |
| CV0073_Covid_NK.16hi | 68 | 364 | Healthy |
| CV0074_Covid_NK.16hi | 101 | 120 | Healthy |
| CV0084_Covid_NK.16hi | 216 | 153 | Covid |
| CV0094_Covid_NK.16hi | 350 | 0 | Covid |
| CV0100_Covid_NK.16hi | 133 | 113 | Covid |
| CV0104_Covid_NK.16hi | 252 | 24 | Covid |
| CV0120_Covid_NK.16hi | 258 | 18 | Covid |
| CV0128_Covid_NK.16hi | 47 | 107 | Healthy |
| CV0134_Covid_NK.16hi | 58 | 5 | Covid |
| CV0137_Covid_NK.16hi | 269 | 481 | Healthy |
| CV0144_Covid_NK.16hi | 17 | 22 | Healthy |
| CV0155_Covid_NK.16hi | 94 | 82 | Covid |
| CV0160_Covid_NK.16hi | 132 | 40 | Covid |
| CV0164_Covid_NK.16hi | 328 | 196 | Covid |
| CV0171_Covid_NK.16hi | 672 | 100 | Covid |
| CV0176_Covid_NK.16hi | 259 | 160 | Covid |
| CV0178_Covid_NK.16hi | 339 | 801 | Healthy |
| CV0180_Covid_NK.16hi | 39 | 32 | Covid |
| CV0198_Covid_NK.16hi | 3 | 6 | Healthy |
| CV0200_Covid_NK.16hi | 27 | 28 | Healthy |
| CV0201_Covid_NK.16hi | 175 | 45 | Covid |
| CV0231_Covid_NK.16hi | 282 | 100 | Covid |
| CV0234_Covid_NK.16hi | 305 | 58 | Covid |
| CV0257_Covid_NK.16hi | 297 | 170 | Covid |
| CV0262_Covid_NK.16hi | 217 | 221 | Healthy |
| CV0279_Covid_NK.16hi | 113 | 152 | Healthy |
| CV0284_Covid_NK.16hi | 377 | 44 | Covid |
| CV0326_Covid_NK.16hi | 232 | 16 | Covid |
| CV0902_Healthy_NK.16hi | 226 | 549 | Healthy |
| CV0904_Healthy_NK.16hi | 60 | 601 | Healthy |
| CV0911_Healthy_NK.16hi | 94 | 475 | Healthy |
| CV0915_Healthy_NK.16hi | 92 | 123 | Healthy |
| CV0917_Healthy_NK.16hi | 48 | 148 | Healthy |
| CV0926_Healthy_NK.16hi | 70 | 282 | Healthy |
| CV0929_Healthy_NK.16hi | 31 | 42 | Healthy |
| CV0934_Healthy_NK.16hi | 39 | 164 | Healthy |
| CV0939_Healthy_NK.16hi | 112 | 71 | Covid |
| CV0940_Healthy_NK.16hi | 107 | 135 | Healthy |
| CV0944_Healthy_NK.16hi | 70 | 77 | Healthy |
| MH8919176_Healthy_NK.16hi | 10 | 335 | Healthy |
| MH8919177_Healthy_NK.16hi | 5 | 269 | Healthy |
| MH8919178_Healthy_NK.16hi | 19 | 608 | Healthy |
| MH8919179_Healthy_NK.16hi | 17 | 457 | Healthy |
| MH8919226_Healthy_NK.16hi | 6 | 235 | Healthy |
| MH8919227_Healthy_NK.16hi | 105 | 603 | Healthy |
| MH8919282_Healthy_NK.16hi | 97 | 1020 | Healthy |
| MH8919283_Healthy_NK.16hi | 179 | 430 | Healthy |
| MH8919326_Covid_NK.16hi | 1196 | 10 | Covid |
| MH8919327_Covid_NK.16hi | 2310 | 21 | Covid |
| MH8919328_Covid_NK.16hi | 587 | 161 | Covid |
| MH8919329_Covid_NK.16hi | 1108 | 127 | Covid |
| MH8919330_Covid_NK.16hi | 1288 | 158 | Covid |
| MH8919331_Covid_NK.16hi | 1190 | 62 | Covid |
| MH8919332_Healthy_NK.16hi | 140 | 886 | Healthy |
| MH8919333_Healthy_NK.16hi | 230 | 1419 | Healthy |
| MH9143270_Covid_NK.16hi | 550 | 19 | Covid |
| MH9143271_Covid_NK.16hi | 701 | 152 | Covid |
| MH9143272_Covid_NK.16hi | 1513 | 117 | Covid |
| MH9143273_Covid_NK.16hi | 469 | 45 | Covid |
| MH9143274_Covid_NK.16hi | 203 | 104 | Covid |
| MH9143275_Covid_NK.16hi | 262 | 0 | Covid |
| MH9143276_Covid_NK.16hi | 690 | 154 | Covid |
| MH9143277_Covid_NK.16hi | 378 | 67 | Covid |
| MH9143320_Covid_NK.16hi | 2500 | 13 | Covid |
| MH9143321_Covid_NK.16hi | 679 | 777 | Healthy |
| MH9143322_Covid_NK.16hi | 1437 | 37 | Covid |
| MH9143323_Covid_NK.16hi | 1854 | 9 | Covid |
| MH9143324_Covid_NK.16hi | 946 | 448 | Covid |
| MH9143325_Covid_NK.16hi | 2365 | 46 | Covid |
| MH9143326_Covid_NK.16hi | 1352 | 154 | Covid |
| MH9143327_Covid_NK.16hi | 2274 | 225 | Covid |
| MH9143371_Covid_NK.16hi | 638 | 22 | Covid |
| MH9143420_Covid_NK.16hi | 333 | 276 | Covid |
| MH9143421_Covid_NK.16hi | 2340 | 22 | Covid |
| MH9143422_Covid_NK.16hi | 1457 | 167 | Covid |
| MH9143423_Covid_NK.16hi | 2021 | 26 | Covid |
| MH9143424_Covid_NK.16hi | 1838 | 31 | Covid |
| MH9143425_Covid_NK.16hi | 756 | 58 | Covid |
| MH9143426_Covid_NK.16hi | 1424 | 304 | Covid |
| MH9143427_Covid_NK.16hi | 2853 | 188 | Covid |
| MH9179821_Covid_NK.16hi | 1661 | 9 | Covid |
| MH9179822_Covid_NK.16hi | 1488 | 30 | Covid |
| MH9179823_Covid_NK.16hi | 577 | 76 | Covid |
| MH9179824_Covid_NK.16hi | 637 | 177 | Covid |
| MH9179825_Covid_NK.16hi | 627 | 57 | Covid |
| MH9179826_Covid_NK.16hi | 410 | 35 | Covid |
| newcastle004v2_Covid_NK.16hi | 1628 | 227 | Covid |
| newcastle20_Covid_NK.16hi | 178 | 69 | Covid |
| newcastle21_Covid_NK.16hi | 1382 | 150 | Covid |
| newcastle21v2_Covid_NK.16hi | 1151 | 332 | Covid |
| newcastle49_Covid_NK.16hi | 319 | 5 | Covid |
| newcastle59_Covid_NK.16hi | 523 | 11 | Covid |
| newcastle65_Healthy_NK.16hi | 51 | 501 | Healthy |
| newcastle74_Healthy_NK.16hi | 330 | 1072 | Healthy |

Table S9 GRAD-CAM [KEGG 2026](https://maayanlab.cloud/Enrichr/enrich) pathway enrichment analysis results

| **Index** | **Name** | **P-value** | **Adjusted p-value** | **Odds Ratio** | **Combined score** |
| --- | --- | --- | --- | --- | --- |
| 1 | PROTEIN PROCESSING IN ENDOPLASMIC RETICULUM | 0.0003708 | 0.01372 | 13.17 | 104.03 |
| 2 | RENIN SECRETION | 0.008408 | 0.1312 | 15.63 | 74.67 |
| 3 | RNA DEGRADATION | 0.01064 | 0.1312 | 13.77 | 62.56 |
| 4 | INFLAMMATORY MEDIATOR REGULATION OF TRP CHANNELS | 0.01611 | 0.1490 | 11.01 | 45.43 |
| 5 | FATTY ACID BIOSYNTHESIS | 0.03541 | 0.2424 | 30.08 | 100.49 |
| 6 | CELL CYCLE | 0.03932 | 0.2424 | 6.72 | 21.76 |
| 7 | FOLATE BIOSYNTHESIS | 0.05265 | 0.2783 | 19.66 | 57.88 |
| 8 | PROTEIN EXPORT | 0.06022 | 0.2785 | 17.03 | 47.86 |
| 9 | PRIMARY IMMUNODEFICIENCY | 0.07332 | 0.3014 | 13.81 | 36.08 |
| 10 | STEROID HORMONE BIOSYNTHESIS | 0.1187 | 0.3984 | 8.23 | 17.54 |

Table S10 GRAD-CAM MSigDB Hallmark 2020 pathway enrichment analysis results

| **Index** | **Name** | **P-value** | **Adjusted p-value** | **Odds Ratio** | **Combined score** |
| --- | --- | --- | --- | --- | --- |
| 1 | IL-2/STAT5 Signaling | 0.0006566 | 0.01116 | 11.26 | 82.53 |
| 2 | Unfolded Protein Response | 0.02146 | 0.1824 | 9.41 | 36.15 |
| 3 | TNF-alpha Signaling via NF-kB | 0.06057 | 0.2574 | 5.25 | 14.73 |
| 4 | Inflammatory Response | 0.06057 | 0.2574 | 5.25 | 14.73 |
| 5 | Androgen Response | 0.1818 | 0.3313 | 5.14 | 8.77 |
| 6 | Coagulation | 0.2421 | 0.3313 | 3.71 | 5.26 |
| 7 | Fatty Acid Metabolism | 0.2721 | 0.3313 | 3.23 | 4.21 |
| 8 | Apoptosis | 0.2765 | 0.3313 | 3.17 | 4.08 |
| 9 | G2-M Checkpoint | 0.3313 | 0.3313 | 2.55 | 2.81 |
| 10 | heme Metabolism | 0.3313 | 0.3313 | 2.55 | 2.81 |

Table S11 GRAD-CAM GO Biological Process 2025 ontology enrichment analysis results

| **Index** | **Name** | **P-value** | **Adjusted p-value** | **Odds Ratio** | **Combined score** |
| --- | --- | --- | --- | --- | --- |
| 1 | Establishment of Protein Localization to Endoplasmic Reticulum (GO:0072599) | 0.0004598 | 0.1006 | 74.98 | 576.24 |
| 2 | ERK1 and ERK2 Cascade (GO:0070371) | 0.0008784 | 0.1006 | 52.47 | 369.28 |
| 3 | T-helper Cell Differentiation (GO:0042093) | 0.0009608 | 0.1006 | 49.97 | 347.19 |
| 4 | Positive Regulation of Inflammatory Response (GO:0050729) | 0.001567 | 0.1230 | 14.37 | 92.80 |
| 5 | Positive Regulation of Defense Response (GO:0031349) | 0.002224 | 0.1235 | 12.66 | 77.35 |
| 6 | Positive Regulation of Response to External Stimulus (GO:0032103) | 0.004209 | 0.1235 | 10.03 | 54.89 |
| 7 | Protein Metabolic Process (GO:0019538) | 0.007266 | 0.1235 | 4.54 | 22.35 |
| 8 | Positive Regulation of Apoptotic Signaling Pathway (GO:2001235) | 0.008644 | 0.1235 | 15.40 | 73.15 |
| 9 | Epithelial Tube Branching Involved in Lung Morphogenesis (GO:0060441) | 0.009961 | 0.1235 | 127.92 | 589.61 |
| 10 | Regulation of Blood Vessel Remodeling (GO:0060312) | 0.009961 | 0.1235 | 127.92 | 589.61 |

Table S12 GRAD-CAM GO Molecular Function 2025 ontology enrichment analysis results

| **Index** | **Name** | **P-value** | **Adjusted p-value** | **Odds Ratio** | **Combined score** |
| --- | --- | --- | --- | --- | --- |
| 1 | Calcium Ion Transmembrane Transporter Activity (GO:0015085) | 0.009619 | 0.1063 | 14.54 | 67.51 |
| 2 | Sulfatide Binding (GO:0120146) | 0.009961 | 0.1063 | 127.92 | 589.61 |
| 3 | Arachidonate Binding (GO:0050544) | 0.01194 | 0.1063 | 102.33 | 453.10 |
| 4 | Icosatetraenoic Acid Binding (GO:0050543) | 0.01194 | 0.1063 | 102.33 | 453.10 |
| 5 | Icosanoid Binding (GO:0050542) | 0.01392 | 0.1063 | 85.27 | 364.51 |
| 6 | G-quadruplex RNA Binding (GO:0002151) | 0.01392 | 0.1063 | 85.27 | 364.51 |
| 7 | Calcium Channel Activity (GO:0005262) | 0.01611 | 0.1063 | 11.01 | 45.43 |
| 8 | Prostaglandin Receptor Activity (GO:0004955) | 0.01786 | 0.1063 | 63.95 | 257.41 |
| 9 | RAGE Receptor Binding (GO:0050786) | 0.01786 | 0.1063 | 63.95 | 257.41 |
| 10 | G-quadruplex DNA Binding (GO:0051880) | 0.01786 | 0.1063 | 63.95 | 257.41 |

Table S13 GRAD-CAM Reactome Pathways 2024 pathway enrichment analysis results

| **Index** | **Name** | **P-value** | **Adjusted p-value** | **Odds Ratio** | **Combined score** |
| --- | --- | --- | --- | --- | --- |
| 1 | Transcription of E2F Targets Under Neg Control by P107 (RBL1) and P130 (RBL2) in Complex With HDAC1 | 0.0004598 | 0.03612 | 74.98 | 576.24 |
| 2 | TP53 Regulates Transcription of Genes Involved in G2 Cell Cycle Arrest | 0.0005847 | 0.03612 | 65.61 | 488.39 |
| 3 | Transcription of E2F Targets Under Negative Control by DREAM Complex | 0.0006527 | 0.03612 | 61.74 | 452.85 |
| 4 | G0 and Early G1 | 0.001326 | 0.05080 | 41.97 | 278.06 |
| 5 | G1 S-Specific Transcription | 0.001530 | 0.05080 | 38.86 | 251.88 |
| 6 | SMAD2 SMAD3 SMAD4 Heterotrimer Regulates Transcription | 0.002354 | 0.05968 | 30.85 | 186.67 |
| 7 | Neutrophil Degranulation | 0.002521 | 0.05968 | 5.89 | 35.21 |
| 8 | Cyclin D Associated Events in G1 | 0.003983 | 0.05968 | 23.29 | 128.71 |
| 9 | G1 Phase | 0.003983 | 0.05968 | 23.29 | 128.71 |
| 10 | XBP1(S) Activates Chaperone Genes | 0.004322 | 0.05968 | 22.30 | 121.40 |

Table S14 SHAP Reactome Pathways 2024 pathway enrichment analysis results

| **Index** | **Name** | **P-value** | **Adjusted p-value** | **Odds Ratio** | **Combined score** |
| --- | --- | --- | --- | --- | --- |
| 1 | Interleukin-4 and Interleukin-13 Signaling | 0.001452 | 0.1737 | 14.77 | 96.49 |
| 2 | Phosphorylation of CD3 and TCR Zeta Chains | 0.001530 | 0.1737 | 38.86 | 251.88 |
| 3 | TFAP2 (AP-2) Family Regulates Transcription of Cell Cycle Factors | 0.009961 | 0.2250 | 127.92 | 589.61 |
| 4 | MAPK6 MAPK4 Signaling | 0.01117 | 0.2250 | 13.42 | 60.30 |
| 5 | Mitochondrial Translation Elongation | 0.01428 | 0.2250 | 11.75 | 49.93 |
| 6 | Mitochondrial Translation Initiation | 0.01428 | 0.2250 | 11.75 | 49.93 |
| 7 | Mitochondrial Translation Termination | 0.01428 | 0.2250 | 11.75 | 49.93 |
| 8 | Binding of TCF LEF CTNNB1 to Target Gene Promoters | 0.01589 | 0.2250 | 73.09 | 302.73 |
| 9 | Insulin-like Growth Factor-2 mRNA Binding Proteins (IGF2BPs IMPs VICKZs) Bind RNA | 0.01589 | 0.2250 | 73.09 | 302.73 |
| 10 | DNA Replication Initiation | 0.01589 | 0.2250 | 73.09 | 302.73 |

Table S15 SHAP KEGG Pathways 2026 pathway enrichment analysis results

| **Index** | **Name** | **P-value** | **Adjusted p-value** | **Odds Ratio** | **Combined score** |
| --- | --- | --- | --- | --- | --- |
| 1 | PHOSPHONATE AND PHOSPHINATE METABOLISM | 0.01194 | 0.3178 | 102.33 | 453.10 |
| 2 | SMALL CELL LUNG CANCER | 0.01457 | 0.3178 | 11.62 | 49.13 |
| 3 | SPLICEOSOME | 0.03359 | 0.3178 | 7.35 | 24.93 |
| 4 | CELL ADHESION MOLECULE (CAM) INTERACTION | 0.03841 | 0.3178 | 6.81 | 22.21 |
| 5 | RIBOSOME | 0.04069 | 0.3178 | 6.60 | 21.12 |
| 6 | INFLUENZA A | 0.04586 | 0.3178 | 6.16 | 19.00 |
| 7 | ASTHMA | 0.05833 | 0.3178 | 17.62 | 50.08 |
| 8 | PROTEOGLYCANS IN CANCER | 0.05898 | 0.3178 | 5.33 | 15.10 |
| 9 | EPSTEIN-BARR VIRUS INFECTION | 0.06218 | 0.3178 | 5.17 | 14.37 |
| 10 | DNA REPLICATION | 0.06773 | 0.3178 | 15.03 | 40.46 |

Table S16 SHAP MSigDB Hallmark 2020 pathway enrichment analysis results

| **Index** | **Name** | **P-value** | **Adjusted p-value** | **Odds Ratio** | **Combined score** |
| --- | --- | --- | --- | --- | --- |
| 1 | IL-2/STAT5 Signaling | 0.0006566 | 0.002676 | 11.26 | 82.53 |
| 2 | G2-M Checkpoint | 0.0006691 | 0.002676 | 11.20 | 81.90 |
| 3 | Estrogen Response Early | 0.0006691 | 0.002676 | 11.20 | 81.90 |
| 4 | Allograft Rejection | 0.0006691 | 0.002676 | 11.20 | 81.90 |
| 5 | E2F Targets | 0.0006691 | 0.002676 | 11.20 | 81.90 |
| 6 | Myc Targets V1 | 0.0006691 | 0.002676 | 11.20 | 81.90 |
| 7 | UV Response Up | 0.03977 | 0.1211 | 6.68 | 21.55 |
| 8 | TNF-alpha Signaling via NF-kB | 0.06057 | 0.1211 | 5.25 | 14.73 |
| 9 | Estrogen Response Late | 0.06057 | 0.1211 | 5.25 | 14.73 |
| 10 | Interferon Gamma Response | 0.06057 | 0.1211 | 5.25 | 14.73 |

Table S17 SHAP GO Biological Process 2025 ontology enrichment analysis results

| **Index** |  | **Name** | **P-value** | **Adjusted p-value** | **Odds Ratio** | **Combined score** |
| --- | --- | --- | --- | --- | --- | --- |
| 1 |  | Actin Crosslink Formation (GO:0051764) | 0.02570 | 0.1295 | 42.62 | 156.06 |
| 2 |  | Actin Filament Bundle Assembly (GO:0051017) | 0.07146 | 0.1558 | 14.19 | 37.44 |
| 3 |  | Actin Filament Bundle Organization (GO:0061572) | 0.07332 | 0.1579 | 13.81 | 36.08 |
| 4 |  | Actin Filament Organization (GO:0007015) | 0.2938 | 0.3279 | 2.95 | 3.61 |
| 5 |  | Actin Filament Polymerization (GO:0030041) | 0.05455 | 0.1459 | 18.93 | 55.06 |
| 6 |  | Actin Polymerization or Depolymerization (GO:0008154) | 0.09718 | 0.1750 | 10.21 | 23.80 |
| 7 |  | Ameboidal-type Cell Migration (GO:0001667) | 0.04311 | 0.1381 | 24.35 | 76.54 |
| 8 |  | Anterograde Trans-Synaptic Signaling (GO:0098916) | 0.04780 | 0.1440 | 6.02 | 18.30 |
| 9 |  | Antigen Processing and Presentation of Exogenous Peptide Antigen (GO:0002478) | 0.06959 | 0.1558 | 14.60 | 38.90 |
| 10 |  | Antigen Processing and Presentation of Exogenous Peptide Antigen via MHC Class II (GO:0019886) | 0.05265 | 0.1459 | 19.66 | 57.88 |

Table S18 SHAP GO Cellular Component 2025 ontology enrichment analysis results

| **Index** | **Name** | **P-value** | **Adjusted p-value** | **Odds Ratio** | **Combined score** |
| --- | --- | --- | --- | --- | --- |
| 1 | Cytoplasmic Vesicle Membrane (GO:0030659) | 0.00002974 | 0.002617 | 9.22 | 96.08 |
| 2 | Bounding Membrane of Organelle (GO:0098588) | 0.0005419 | 0.02384 | 4.98 | 37.46 |
| 3 | Late Endosome (GO:0005770) | 0.001399 | 0.04104 | 9.13 | 60.00 |
| 4 | Multivesicular Body (GO:0005771) | 0.004674 | 0.1028 | 21.39 | 114.76 |
| 5 | Slit Diaphragm (GO:0036057) | 0.009961 | 0.1179 | 127.92 | 589.61 |
| 6 | Bleb (GO:0032059) | 0.009961 | 0.1179 | 127.92 | 589.61 |
| 7 | Filtration Diaphragm (GO:0036056) | 0.009961 | 0.1179 | 127.92 | 589.61 |
| 8 | Actin Filament (GO:0005884) | 0.01198 | 0.1179 | 12.92 | 57.15 |
| 9 | Vesicle Membrane (GO:0012506) | 0.01226 | 0.1179 | 12.76 | 56.16 |
| 10 | Intracellular Membrane-Bounded Organelle (GO:0043231) | 0.01339 | 0.1179 | 2.16 | 9.30 |

Table S19 SHAP GO Molecular Function 2025 ontology enrichment analysis results

| **Index** | **Name** | **P-value** | **Adjusted p-value** | **Odds Ratio** | **Combined score** |
| --- | --- | --- | --- | --- | --- |
| 1 | Endopeptidase Inhibitor Activity (GO:0004866) | 0.002128 | 0.1038 | 12.87 | 79.16 |
| 2 | Peptidase Inhibitor Activity (GO:0030414) | 0.003344 | 0.1038 | 25.57 | 145.76 |
| 3 | Protein Serine/Threonine Kinase Activator Activity (GO:0043539) | 0.008408 | 0.1038 | 15.63 | 74.67 |
| 4 | ATP:ADP Antiporter Activity (GO:0005471) | 0.009961 | 0.1038 | 127.92 | 589.61 |
| 5 | Oxidoreductase Activity, Acting on a Sulfur Group of Donors, Disulfide as Acceptor (GO:0016671) | 0.01194 | 0.1038 | 102.33 | 453.10 |
| 6 | CDP-alcohol Phosphatidyltransferase Activity (GO:0017169) | 0.01194 | 0.1038 | 102.33 | 453.10 |
| 7 | Minor Groove of Adenine-Thymine-Rich DNA Binding (GO:0003680) | 0.01392 | 0.1038 | 85.27 | 364.51 |
| 8 | Peroxisome Proliferator Activated Receptor Binding (GO:0042975) | 0.01392 | 0.1038 | 85.27 | 364.51 |
| 9 | Preribosome Binding (GO:1990275) | 0.01589 | 0.1038 | 73.09 | 302.73 |
| 10 | 5'-Deoxyribose-5-Phosphate Lyase Activity (GO:0051575) | 0.01589 | 0.1038 | 73.09 | 302.73 |
